# Robust fisheries management strategies under deep uncertainty

**DOI:** 10.1038/s41598-024-68006-5

**Published:** 2024-07-23

**Authors:** Jan Conradt, Steffen Funk, Camilla Sguotti, Rudi Voss, Thorsten Blenckner, Christian Möllmann

**Affiliations:** 1 Institute of Marine Ecosystem and Fishery Science, Universität Hamburg, Große Elbstraße 133, 22767 Hamburg, Germany; 2 Department of Biology, University of Padova, Via U. Bassi 58/B, 85121 Padova, Italy; 3 German Centre for Integrative Biodiversity Research (iDiv), Puschstraße 4, 04103 Leipzig, Germany; 4 Center for Ocean and Society (CeOS), Christian-Albrechts-University Kiel, Neufeldtstraße 10, 24118 Kiel, Germany; 5 Stockholm Resilience Centre, Stockholm University, Frescativägen 8, 10691 Stockholm, Sweden

**Keywords:** Fisheries, Sustainability

## Abstract

Fisheries worldwide face uncertain futures as climate change manifests in environmental effects of hitherto unseen strengths. Developing climate-ready management strategies traditionally requires a good mechanistic understanding of stock response to climate change in order to build projection models for testing different exploitation levels. Unfortunately, model-based projections of fish stocks are severely limited by large uncertainties in the recruitment process, as the required stock-recruitment relationship is usually not well represented by data. An alternative is to shift focus to improving the decision-making process, as postulated by the decision-making under deep uncertainty (DMDU) framework. Robust Decision Making (RDM), a key DMDU concept, aims at identifying management decisions that are robust to a vast range of uncertain scenarios. Here we employ RDM to investigate the capability of North Sea cod to support a sustainable and economically viable fishery under future climate change. We projected the stock under 40,000 combinations of exploitation levels, emission scenarios and stock-recruitment parameterizations and found that model uncertainties and exploitation have similar importance for model outcomes. Our study revealed that no management strategy exists that is fully robust to the uncertainty in relation to model parameterization and future climate change. We instead propose a risk assessment that accounts for the trade-offs between stock conservation and profitability under deep uncertainty.

## Introduction

Fisheries worldwide face uncertain futures as climate change manifests in environmental effects of hitherto unseen strengths^1,2^. Developing climate-resilient management strategies traditionally requires well-supported mechanistic hypotheses of how fish stocks respond to the effects of climate change in order to build projection models to be tested with different degrees of exploitation^3^. Model-based projections of marine social-ecological systems including fisheries are however notoriously impeded by uncertainty about key ecological processes^4–6^. Such uncertainty often arises from limitations in the understanding of their intricate mechanisms and their relationships to physical variables like temperature. Resulting simplified models reflect a general consensus about the most basic mechanisms, e.g. models describing larval dispersal contain well-known hydrodynamic processes but not poorly-understood effects of larval behaviour (e.g.^7^). In fisheries science, a major challenge is the prediction of the strength of the incoming year-class as a basis for setting future fishing opportunities for the industry^3,8^. This “recruitment” process is the result of a multitude of complex biological processes such as growth-rate variability^9,10^ and physical processes like larval drift^11–13^. Prediction of the number of incoming offspring is hence usually based on the assumption that the size of the mature population, the spawning stock biomass (SSB), is the main predictor^14^. The nature of mechanisms that go beyond this most basic assumption, such as the importance of environmental variability or the role of feedback effects of recruitment on SSB^15^, are subject to debate (e.g.^3,16^). Hence, lacking ecological understanding and limited data quality and quantity cause the existence of multiple interpretations about the responsible factors and the functional forms of these “stock-recruitment” (SR) relationships.

The inability to agree on the mechanisms behind critical processes in a dynamic system is a key characteristic of the theoretical concept of “Deep Uncertainty”^17^. In the decision-making literature, Deep Uncertainty (DU) is considered to be the strongest level of uncertainty (e.g.^18,19^). DU is characterized by situations in which experts are unable to find intellectual consensus on the mechanisms behind system processes, where a quantification of uncertainty (e.g. in the form of probability distributions) is not possible, or where unpredictable events are known to occur^20^. With respect to forecasting this means that the number of scenarios to be considered would be large and not necessarily limited to a few discrete instances. In contrast to DU, lower levels of uncertainty are characterized by either the possibility to predict probabilistically (i.e. based on probability density or on different levels of plausibility) or by the possibility to formulate a low number of discrete, equally plausible futures^20^.

DU is increasingly considered in projections of management systems expected to become severely affected by climate change, e.g.in water management^21^ and ski resorts^22^. However, modeling of ecological systems and population modeling tends to ignore the existence of this strong uncertainty level. For example, Management Strategy Evaluation (MSE), an extended version of modeling fisheries systems under various candidate management strategies, usually performs projections under several scenarios that are assigned a plausibility rank. This rank is based on expert knowledge, and the scenario outcomes are weighted based on plausibility in order to assess the vulnerability of the management strategy candidates^23^. Within MSE, but also in stock projections in general, recruitment of fish stocks is often projected via statistical parameter estimates of the SR model to which residuals from the observations are added randomly (e.g.^24^). The usage of the mean SR model parameter estimates often assumes that recruitment uncertainty can be characterized by probability. Such an approach can be considered as an example of an “expected-utility framework”, characterizing decision-making approaches where scenarios are assigned subjective probabilities^25^.

Yet there are clear indications that working with plausibilities and probabilities have limitations in applied modeling like MSE, because it is often difficult to find consensus on the plausibility of a certain scenario^23^. Consequently, fish stock dynamics are likely subject to higher levels of uncertainty than currently recognized, which can limit the utility of the MSE approach that is more constrained by i.a. relatively high complexity and related data dependency^26^. Furthermore, such uncertainty is not simply due to lacking knowledge, but of ambiguous nature that is symptomatic to DU problems, and may lead to poor decision-making caused by narrow-focused analyses^27^. Howell et al*.*^28^ recognized this problem, found the uncertainty in population size projected under different SR hypotheses to be “unquantifiable”, an attribute of DU^20^, and proposed a wide range of scenarios to perform MSE with.^29^ characterized the ignorance of DU as a major concern in long-term planning of ecosystem management, including fisheries management, and advocated to widen the range of uncertainty considered and the development of strategies robust against it.

The science of dealing with such high-level uncertainties, formally known as “Decision-Making under Deep Uncertainty” (DMDU), has seen the development of a number of concepts that address the difficulty in performing precise projections from a practical, management-based point-of-view^20^. The most popular of these is the exploration-based “Robust Decision Making” (RDM) used to analyze and stress-test candidate management strategies^30,31^. Other DMDU approaches are Dynamic Adaptive Planning^32,33^ and Dynamic Adaptive Policy Pathways^34^, which focus on specifying rules for decision adaptation over time or the prior formulation and evaluation of alternative decision routes.

Common to all DMDU approaches, but to RDM in particular, is the proposition to shift emphasis from improving model predictions to improving management decisions^25^. This proposition is based on the observation that improving predictions often involves increasing model complexity, which in turn increases the number of uncertain factors, and that better predictive capability does not necessarily result in better decision-making^35^. The aim of RDM is thus to increase an understanding about the consequences of management actions under a large spectrum of possible scenarios, and to help define a management strategy that achieves the desired outcomes under DU, i.e. is robust to a multitude of different but equally possible futures^36^. To this end, RDM employs the generation of a large number of model projection runs for each candidate management strategy. Each run represents one uncertain scenario; these scenarios can include discrete scenarios, such sampled from a continuous range or a combination thereof. Results from these runs are then aggregated and investigated using e.g. Machine-Learning or visualization tools to (i) determine the importance of uncertain parameters in achieving management objectives (exploratory modeling), (ii) determine conditions under which a candidate strategy fails or succeeds (scenario discovery) and (iii) unveil potential trade-offs between multiple objectives^31^. Insights yielded from these analyses are often used to update management strategy candidates, which are then again subjected to modeling under the same range of uncertain scenarios. Once the RDM analyses are completed, a candidate strategy that fulfils the desired outcomes to the greatest extent possible under the largest number of scenarios is chosen for implementation^30^.

The consideration of DU and the usage of DMDU methods have been explicitly proposed for fisheries management^37,38^, though RDM has as yet not been put into applied use in the research field. Here we apply the RDM framework to uncover robust management strategies for North Sea cod (*Gadus morhua* L.) under future climate change. North Sea cod is one of Northern Europe’s most valuable ground-fish stocks, yielding a landings value of approximately 7 billion US$ (1986–2010), with potential economic value under more effective management estimated as approximately 19 billion US$^39^. While historically it was a highly productive resource with catches up to 550 kt estimated for the 1980s^40^, North Sea cod is currently in a low productive state which yields annual catches of 40–50 kt only^41^. The low productive state of North Sea cod is the result of phases of severe overexploitation in the second half of the twentieth century and failed rebuilding attempts in the early twenty-first century^42,43^ which may be the result of climate-driven state shift in productivity^44^ via a negative effect of temperature increase on recruitment^45,46^. With temperature increase expected to continue, and climate effects projected to lead to biomass decreases globally^1,2^, and reorganizations of ecosystems in general^48,49^, sustainable future management is becoming both more complicated and more necessary. Nevertheless, given its economic importance, rebuilding and maintaining North Sea cod is of high importance for the fisheries involved.

We here applied the RDM approach to quantify the potential for both ecologically and economically sustainable management given uncertainties in the recruitment process and the future course of climate change, and to characterize sustainable management strategies. We formulated the results of our study in a risk analysis and trade-off-mapping framework that allowed us to illuminate the potential of sustainably managing North Sea cod under DU.

## Methods

**Figure 1:**
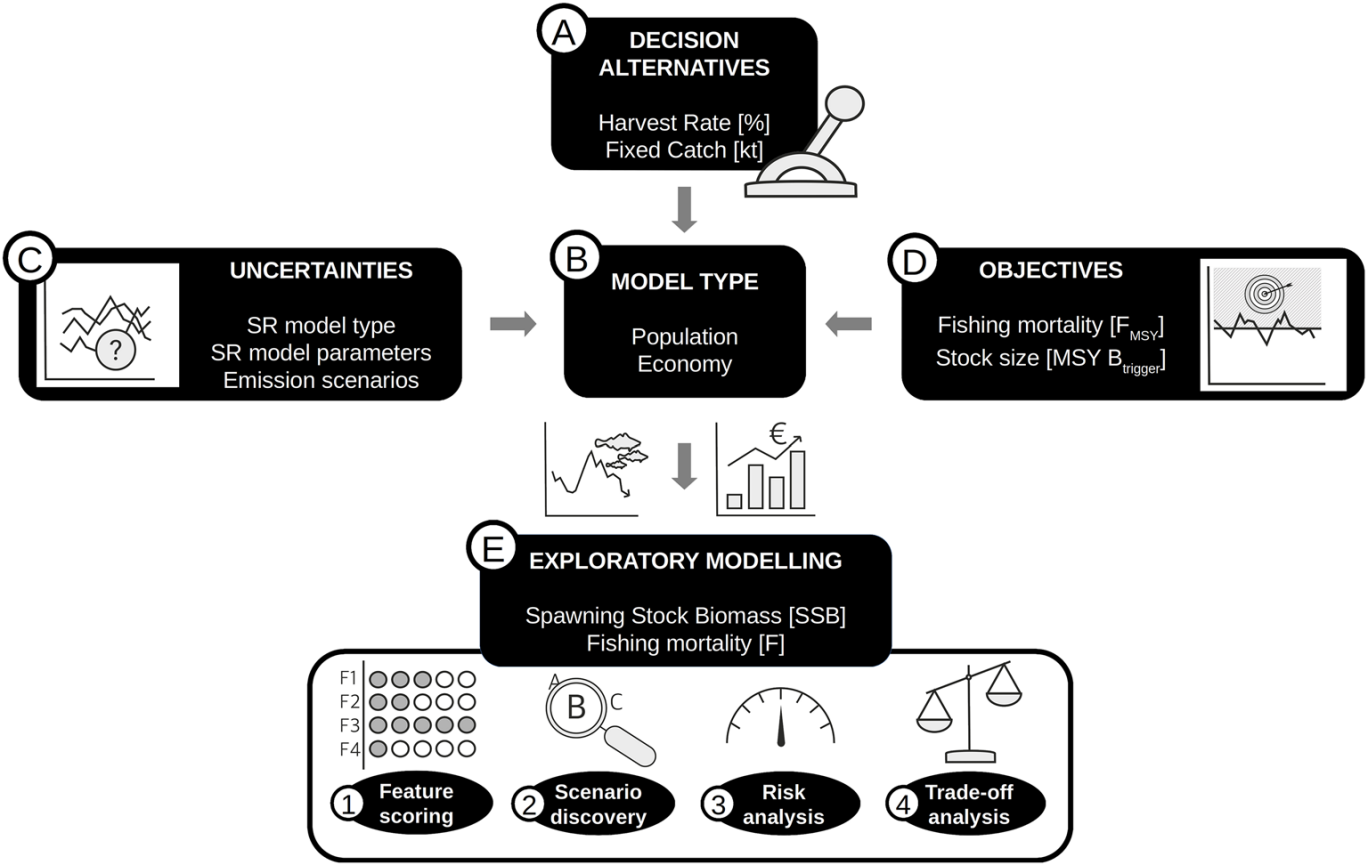
Study design according to Robust Decision Making (RDM) protocols. (**A**) the specification of decision alternatives, i.e. fisheries management strategies according to harvest rates and fixed catch levels; (**B**) the model system consisting of coupled population and economical components; (**C**) uncertainties affecting the success of management strategies, i.e. stock-recruitment (SR) model types and parameterization as well as emission scenarios; (**D**) management objectives that management strategies will be evaluated against; (**E**) exploratory modelling and analysis of model projection outcomes (SSB, fishing mortality [**F**]), including (**1**) evaluation of the relative importance of management measures and uncertainties for achieving objectives (feature scoring), (**2**) identification of combinations of management measures and uncertainties that achieve objectives (scenario discovery) and (**3**) evaluation of the risk of exploitaiton levels not achieving sustainability and profitability objectives (risk analysis), and (**4**) evaluation of trade-offs between exploitation levels as well as sustainability and profitability objectives (trade-off analysis).

Our study follows robust decision making (RDM) protocols^25,31,50^ that consist of (A) identification of the decision-making problem and of decision alternatives, (B) specification of the system structure, i.e. the model used to simulate the effects of management decisions, (C) identification of system uncertainties, (D) development of (potentially conflicting) management objectives, and (E) exploratory modeling (EM) (Fig. 1). EM comprises multiple model projections followed by a multi-way analysis of the simulation results with respect to management objectives^25^.

### Decision alternatives

The decision-making problem in the context of planning long-term fisheries management implies finding exploitation strategies that maintain the stock in a safe biological state while yielding acceptable profits for the fishers who depend on the stock for income^51^. The optimal decision, in accordance with RDM theory, would achieve these aims under a large variety of assumptions about future recruitment dynamics, and would do so under any possible future development of climate change^30^. We here considered two exploitation metrics, i.e. (i) constant catch in tonnes of fish stock biomass, and (ii) constant harvest rate, i.e. a fixed ratio of catch to stock size. Both metrics are used as regulatory metrics in fisheries management to maintain or achieve a safe biological level, but have different advantages and disadvantages^52,53^. Constant catch rules theoretically provide stable catches, but may lead to excessive exploitation rates at low stock sizes. In contrast, catches equal to a fixed proportion of the current stock size (essentially reflecting constant fishing mortality) are more responsive to fluctuations in stock size^54^. In our analysis, decision alternatives for each model run, i.e. the level of catch or the level of harvest rate, were kept constant over all projection years to investigate the long-term viability of each exploitation level.

### Model system

We projected the stock dynamics of North Sea cod for the period 2030–2100 using an age-based single-species population model^55^ where cohorts of equal-aged fish are subject to decrease over time due to fishing, i.e. catch or harvest rate translated to fishing mortality (F), and natural mortality (due to predation and other causes). SSB is calculated as the number of fish per age-class, their age-specific weight and maturity rates. The stock is replenished annually by recruits (age individuals) depending on both the amount of SSB and on environmental pressures. We employed SSB—recruitment (SR) models that include the effect of sea-surface temperature (SST) on offspring production (see below). As a major environmental driver, temperature is frequently applied in the modeling of future management of fisheries (e.g.^56^) and in the design of SR models in particular^57^. We initialized population size at a level equalling MSY B_trigger_ (Supplementary Methods 1) to investigate the impact of DU on management strategies under relatively favourable stock conditions and thus check for potential management challenges beyond stock rebuilding.

Our population model of the North Sea cod stock is coupled to an economic model that computes future profits for the fishery^58^. Profits are based on revenues derived by assigning specific market prices to fish of specific weight, as well as costs. Costs increase with catch, due to e.g. increased requirements for storage capacity and work power. Further details on the population- and economic models are given in the appendix (Supplementary Methods 1 and 4, Supplementary Table 1).

Historical stock data for North Sea cod were obtained from the ICES (International Council for the Exploration of the Sea) Working Group on the Assessment of Demersal Stocks in the North Sea and Skagerrak (WGNSSK^41^). SST observation data for fitting the SR models were retrieved from the NOAA Extended Reconstructed Sea Surface Temperature (ERSST) dataset, version 5^59^. SST projection data were obtained from a regional ocean model^60^, and were bias-corrected against the ERSST data (simple mean bias correction^61^). Pricing data were obtained from the German federal office for agriculture and food^62^.

### Uncertainties

The relationship between SSB, environmental pressures and recruitment is usually subject to strong uncertainty due to the large number of unobserved physical and biological processes involved and the often low amount of high quality data. We hence conducted our RDM analysis around several recruitment scenarios, which were defined by three sources of uncertainty:

#### Functional form of the SR relationship

The relationship between SSB, environmental pressures and recruitment is most commonly modeled via the Ricker^63^ and Beverton–Holt^64^ relationships or their environmentally-sensitive extensions^65,66^. Both models describe initially positive linear effects of SSB, a negative exponential effect of SSB reflecting population and ecosystem capacity limitations and resulting in either asymptotic (Beverton–Holt) or decreasing recruitment (Ricker) at high SSB, and a negative exponential effect of SST (Eq (1); see also Supplementary Methods 1, Supplementary Fig. 1). The high degree of unexplained recruitment variability and lack of recruitment data for very high levels of SSB makes the “true” underlying functional form often unclear^67^. We hence performed our stock projections with both SR models to account for this ambiguity.

1 $$\begin{gathered} R_{t + 1} = N_{t + 1,1} = e^{{ - \gamma E_{t} }} \frac{{\alpha SSB_{t} }}{{1 + \beta SSB_{t} }} \hfill \\ R_{t + 1} = N_{t + 1,1} = \alpha SSB_{t} e^{{ - \beta SSB_{t} - \gamma E_{t} }} \hfill \\ \end{gathered}$$

Equation (1). Environmental Beverton–Holt^66^ (top) and Ricker^65^ (bottom) stock-recruitment-model equation. The strength of the positive linear effect of SSB on recruitment is given by α (recruitment increases with increasing SSB). The limitation of recruitment (or its reduction) through SSB is parameterized by β (ecosystem carrying capacity or other density-related effects like cannibalism). The strength of environmental pressure on recruitment is described by γ. R = recruitment, N = population number, SSB = spawning-stock biomass, E = environmental variable.

We ascertained the adequacy of the environmentally-sensitive SR functions through comparison with a hockey-stick SR function, which is the SR function currently employed by the ICES assessment to describe the SR relationship for North Sea cod^41^, and other climate-insensitive SR functions, in terms of AIC, deviance explained and visual inspection of fit (Supplementary Methods 7).

#### SR model parameterization

SR models only describe very basal assumptions about the effects of SSB and environmental pressures on recruitment, and often fit the data poorly, resulting in wide confidence intervals of parameter estimates^7,57^. In addition to unexplained processes that modify the basal “true” SR relationship, the existence of a singular continuous SR relationship for a given stock itself is challenged by observed “low-recruitment regimes”^68^ and statistical evidence for highly non-linear or discontinuous SR dynamics^45^. We here considered a wide array of continuous SR relationships defined by parameter values sampled from the standard-error range of the statistical estimates (SR equations were re-arranged and logarithms of SSB-related parameters were fitted to avoid sampling biologically meaningless negative parameter values; Supplementary Methods 2). We considered the standard-error range as an estimate of the range of possible SR relationships with equal probability, i.e. the bounds of uniform distributions to sample from (Table 1) (we traded in homoscedascity on the current recruitment time series for covering potential future SR relationships). SR relationships most notably and strongly differed in maximum attainable levels of recruitment (Supplementary Fig. 2). In the context of model projections, this range of SR relationships serves as an expression of the overall deep uncertainty in predicting future recruitment, rather than as a set including one “true” but unknown future SR relationship.

**Table 1:** Sampling bounds for stock-recruitment parameters. Lower and upper bounds are mean parameter estimate ± standard error, respectively.

SR model	Parameter	Lower bound	Upper bound
Ricker	log(alpha)	8.67	12.02
	beta	11.90	12.64
	gamma	0.64	0.95
Beverton–Holt	log(alpha)	9.35	13.23
	beta	− 11.96	− 10.36
	gamma	0.69	1.01

#### Future development of climate change

The future of climate change depends primarily on current and future mitigation measures to reduce carbon emissions^69^. Multiple future pathways of future carbon emissions, the Representative Concentration Pathways (RCP), have been lined out and used to force global and regional climate models that simulate future climate development on a spatial scale^70^. Naturally, implementing climate mitigation measures is not in the purview of fisheries management. Future warming, i.e. an increase of SST, is thus an uncertainty for future recruitment and stock development. We forced the cod population model with projected North Sea SST data for the RCP4.5- and RCP8.5 emissions scenarios, i.e. a “middle-of-the-road” mitigation- and a “business-as-usual” scenario, respectively, through the recruitment process (negative effect of SST on recruitment). These scenarios correspond to different degrees of future SST increases, with increases above the observed maximum occurring more frequently and with a larger magnitude in the latter (Supplementary Methods 3). Data were obtained from a North Sea regional ocean model^60^.

### Objectives

Fisheries management in the European Union applies the Maximum Sustainable Yield (MSY) framework that proposes that under a distinct level of F (i.e. F_MSY_) a stock in safe biological limits can maintain a high level of average catch quasi-indefinitely^71^. Accordingly the MSY concept is the basis against which the International Council for the Exploration of the Sea (ICES) evaluates exploitation and stock status, and gives advice on total allowable catch^71,72^. Management reference points for this approach are the target F, F_MSY_, that theoretically generates MSY, and a precautionary limit biomass level that triggers management action (B_PA_ or MSY B_trigger_) that is used to decrease F at too low biomasses. While both higher and lower F levels will generate lower average yield, exceeding F_MSY_ also puts the stock at risk of decreasing population numbers and F_MSY_ is therefore considered a limit to be avoided^73^. We considered both reference points, i.e. achieving F ≤ F_MSY_ and SSB ≥ MSY B_trigger_ as objectives in our stock simulations. MSY reference points were set to those currently used in the stock assessment of North Sea cod, i.e. F_MSY_ = 0.28 and MSY B_trigger_ = 97.8 kt, which are derived from projections with a climate-insensitive hockey-stick SR model^68^. We consider these reference points from a conservationist perspective, i.e. as limits to overall good stock status (MSY B_trigger_) and acceptable fishing pressure (F_MSY_), and hence do not calculate custom reference points specific to the climate-sensitive SR relationships used in our projections (which, as noted above, have a more expressive rather than true mechanistic meaning). This consideration differs from operational management, where reference points are often adapted to changes in productivity^74^ (as is the case for ICES advice^75^ including North Sea cod^68^).^76^ criticize the operational approach for leading to a lack of precaution under decreasing productivity, and thus implicitly suggest the adoption of a conservationist point-of-view.

### Exploratory modeling

Exploratory Modelling (EM) was conducted by projecting the North Sea cod stock under multiple combinations of uncertain scenarios and management decisions via the climate-forced population model. We initialized the stock in 2030 with a SSB equaling the present MSY B_trigger_ (and corresponding stock numbers, which follow the distribution over age classes estimated for 2018^41^). We thereby assume a successful rebuilding of the presently depleted cod stock until the starting year of the simulation. 40,000 projection runs were conducted consisting of 200 random schemes of SR model parameterizations and climate scenarios, (separate sets of runs for Ricker- and the Beverton & Holt) as well as 100 random management decisions of constant catches and harvest rates (ranges defined based on initial trial simulations; Supplementary Methods 5). Evaluation of projection outcomes was based on procedures commonly applied in EM analysis:

#### Feature scoring

We first evaluated the importance of the various uncertainty factors and the management measures for achieving the management objectives using gradient boosting regression trees^77^. We defined the target regression variable as the number of years in which both management targets, i.e. SSB ≥ MSY B_trigger_ and F ≤ F_MSY_, have been met, and values of the SR parameters and climate scenarios as predictors. Separate regression analyses were performed for each of the Ricker- and the Beverton & Holt SR models.

#### Scenario discovery

In a second step we identified out of all projection runs the successful scenarios where both management targets, i.e. SSB ≥ MSY B_trigger_ and F ≤ F_MSY_, were met for the entire projection period. Subsequently, we explored the combinations of constant catch or harvest rate and uncertain factors that characterize these successful projections.

#### Risk and trade-off analysis

We eventually assessed the risk that different exploitation levels (constant catch levels or harvest rates) will not successfully achieve management objectives. We calculated *sustainability risk* as the risk of F ≥ F_MSY_ (indicating over-fishing^73^) and SSB ≤ MSY B_trigger_ (indicating vulnerability to reproductive failure), and additionally *profitability risk*, reflecting the risk of profit being less than the average profit over the years 2000 to 2018, which is a relatively stable level (i.e. c. 50 million € (model hindcast, see Supplementary Fig. 3)). Risks were calculated as the percentage of projections not meeting at least one of either sustainability objective or not meeting the profitability objective by the total amount of projection data for each management measure.

### Software

All population and economic modelling as well as data analyses were performed in Python^78^. Sampling of uncertainties and decisions in the population model was conducted using the Monte-Carlo sampler of the “EMA Workbench” package for EM tasks^79^. Boosting regression tree analysis was conducted using the “GradientBoostingRegressor” function (with default settings) of the Scikit-Learn package^80^. Visualizations were performed in R^81^ using the “tidyverse” package^82^ and in Python using the “matplotlib” package^83^.

## Results

### Feature scoring

**Figure 2:**
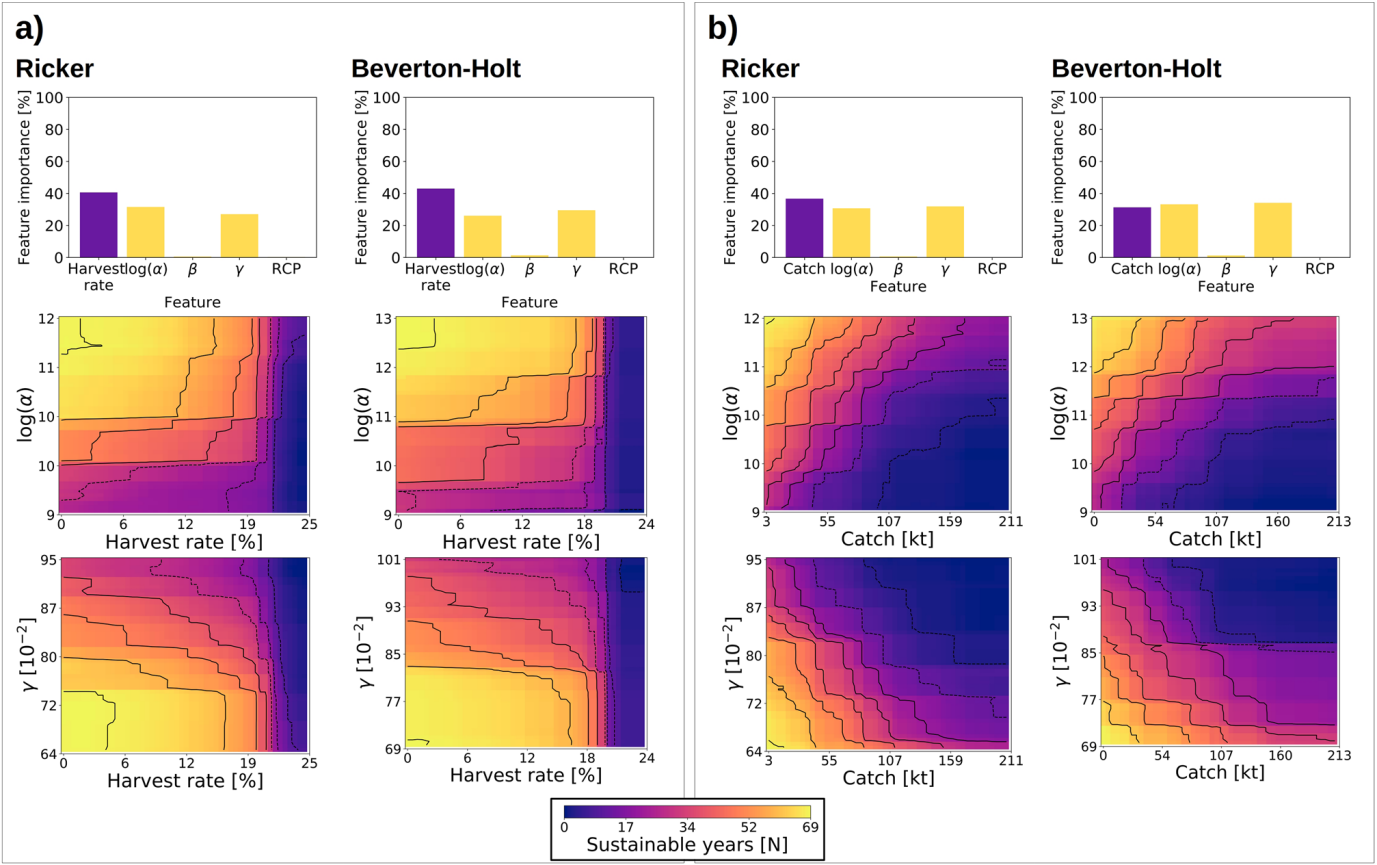
Importance of management measures and uncertainty effects—Results of boosting-regression-tree analysis of projections with Ricker and Beverton–Holt SR-models under harvest rate (**a**) and fixed catch scenarios (**b**); individual effects (upper row) and interactions between management measures and the stock-size-related SR parameter *log(α)* (middle row) and the temperature-related SR parameter *γ* (lower row). Lighter color in interaction plots denotes higher number of sustainable years (i.e. years with SSB ≥ MSY B_trigger_ and F ≤ F_MSY_). RCP = climate scenario (representative concentration pathway).

Feature scoring using boosted regression trees revealed that although exploitation pressure is generally the dominating factor for management success in our simulations of North Sea cod dynamics (except for the combination of the Beverton & Holt model and constant catch), uncertainty in SR model parameters *log(alpha)* and *gamma* is of similar importance (Fig. 2). Our simulations also showed that the realized climate scenario as well as the strength of the density-dependence in the stock (the *log(beta)* parameter in SR model) are likely of minor importance for management success (the number of years in which sustainability objectives are achieved) of North Sea cod. Partial effect plots demonstrate that management success of any of the harvest control rules is dependent on high values of *log(alpha)* (describing the positive effect of SSB on recruitment) and low *gamma* (describing the magnitude of the negative effect of higher SSTs on recruitment) independent of SR model type. In harvest-rate-based management strategies two-dimensional threshold dynamics are clearly visible (Fig. 2a). Thresholds occur between lower and higher management success in relation to *log(alpha)* and *gamma* values, but especially at c. 20% harvest rate to 100% management failure (i.e. zero sustainable years). These are most pronounced at *log(alpha)* levels > c. 10–12 and *gamma* values < c. 0.75–0.80, where almost a full range of future sustainable years is achieved at low harvesting intensity. In contrast, a constant-catch harvest control rule resulted in a more transitional interaction with SR parameter uncertainties (Fig. 2b). Management with harvest rate resulted in a larger safer space of relatively high management success. However, that space is not defined by management strategies alone but also by uncertainty in the SR-model parameterization, in both harvest-control rules.

### Scenario discovery

**Figure 3:**
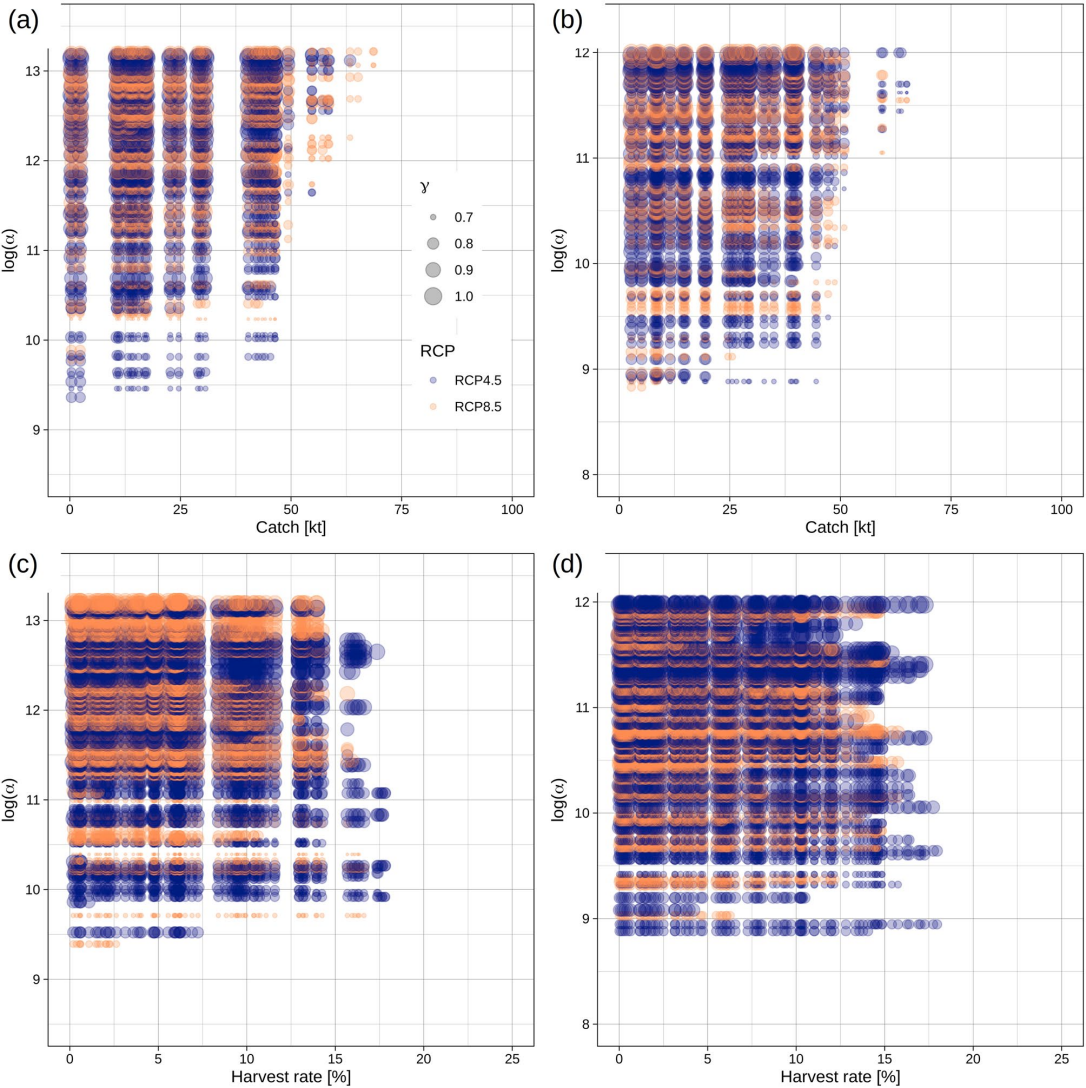
Occurrence of successful scenarios in the policy-uncertainty space—The space is defined by harvest intensity (catch or harvest rate) and the three SR parameters (log(α), log(β) [axis not shown] and γ [shown as dot size]). Successful scenarios are defined as projections with SSB >  = MSY B_trigger_ and F < F_MSY_ in all projection years. Results are shown for Beverton–Holt (**a, c**) and Ricker (**b, d**) SR models under total catch (**a, b**) and harvest rate (**c,d**) scenarios as well as emission scenarios RCP4.5 (blue) and 8.5 (yellow). log(α) and γ (represented by dot size) are SR model parameters.

Scenario discovery revealed that neither a constant catch nor a harvest rate was identifiable that met the sustainability targets over the entire simulation period. Minimum constant catch (0.4 kilo-tonnes) and harvest rates (0.02%) resulted in 68 and 70% successful scenarios, respectively. We found successful scenarios at constant catches < 75 *10^3^ tonnes and harvest rates < c. 18%, with a frequency depending strongly on *log(alpha)* and *gamma* parameters (Fig. 3), a pattern already shown by feature scoring. The highest numbers of successful scenarios were discovered at the lowest catch- and harvest rate levels, but decreased with decreasing *log(alpha)* and increasing *gamma* values. However, the effect of varying *log(alpha)* and *gamma* on the occurrence of successful scenarios is stronger in the constant-catch harvest control rule (Fig. 3a, b) compared to the harvest rate strategy (Fig. 3c, d) that provided a broader safe range of management measures. Successful scenarios are furthermore largely independent of climate scenario and functional form of the SR relationship.

### Risk and trade-off analysis

Our scenario discovery exercise revealed no completely safe levels of catch and harvest rate for North Sea cod given the uncertainty in SR model parameterization; even zero-catch and zero-harvest-rate policies resulted in notable risk (Supplementary Results 2). As a consequence every level of a management measure would bear a degree of risk not achieving the sustainability objectives. We hence assessed the risk that different levels of harvest rates and fixed catches would have on achieving management objectives. In addition to *sustainability risk*, we developed an economic risk metric, i.e. *profitability risk* that indicates the probability that different levels of harvest rates and fixed catches would have to not achieve average recent historical profits. By these metrics we explored the trade-off between risk of not achieving sustainability and the risk of the fishery not operating in a profitable way.

**Figure 4:**
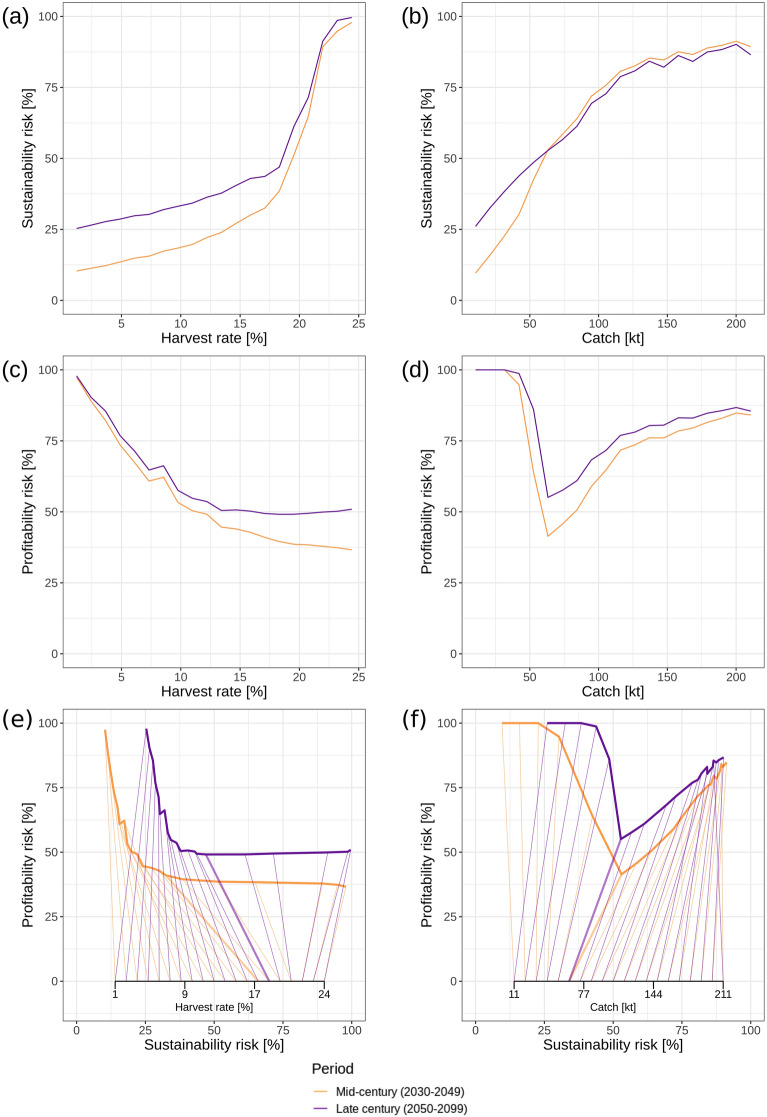
Relationship between sustainability risk and exploitation for harvest-rate (**a**) and fixed-catch projections (**b**), as well as relationship between profitability risk and exploitation (**c**, **d**), and the relationship between sustainability and profitability risks as well as exploitation intensity (inserted x-axis and connecting segments indicate exploitation level associated with a specific risk combination) (**e**, **f**). Risks were calculated over both climate scenarios. Colors represent periods within the projection time series: yellow: 2030–2049 (mid-century), blue: 2050–2099 (end-of-century). Thick segments in (**e**) and (**f**) represent exploitation rates leading to minimum summed risk and a ratio of risks nearest to 1 (see Supplementary Methods 6 for details). For a quantification of risks specific to zero-harvesting scenarios, see Supplementary Results 2.

We found sustainability risk for North Sea cod to slowly increase to 50% towards a harvest rate of c. 20% for both mid- and end-of-century periods, the earlier period however starting from a lower risk level. (Fig. 4a). Afterwards sustainability risk increased faster, approaching 100% at harvest rates of c. 25%. Applying a constant catch harvest control rule would result in a relatively linearly increasing sustainability risk for both periods peaking at c. 80% at a catch of 200 kt (Fig. 4b). Profitability risk decreased continuously with increasing harvest rate levelling off at about 50% (with a slight downward offset for the first period) at the harvest rate causing 100% sustainability risk (Fig. 4c). In contrast, profitability risk decreased abruptly with increasing constant catch from c. 40 kt towards c. 60 kt. From that catch level on profitability risk increased linearly with increasing catch to the peak level causing maximum sustainability risk (Fig. 4d); the increase is likely related on an increase in scenarios that lead to eventual stock collapse and thus to the termination of fishing (Supplementary Results 1).

Our trade-off analysis for harvest rate management strategies revealed an initial rapid decrease of profitability risk (from 100 to c. 50–55%) and a less strong increase in sustainability risk (Fig. 4e) with increasing harvest rates until c. 18%. With a further increase in harvest rates sustainability risk increases rapidly while profitability risk remains constant. An initial steep decrease in profitability risk and an increase in sustainability risk with catches up to c. 63 kt is also found for constant-catch management strategies (Fig. 4f). However, in contrast to harvest rate management, both risks increase in parallel with further increasing catches. Overall both risks are lower for the mid-century compared to the end-of-century period.

**Figure 5:**
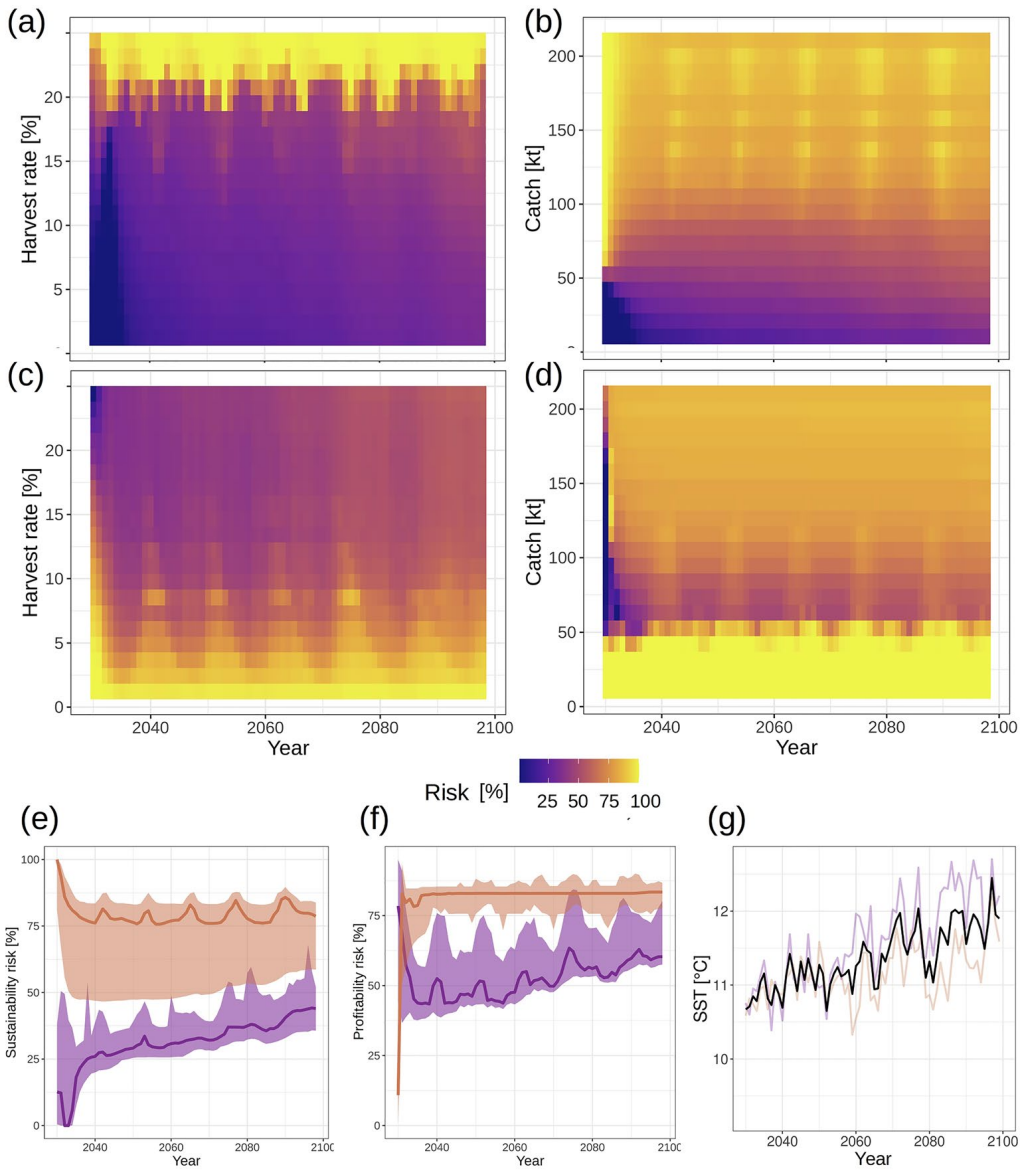
Temporal dynamics of sustainability risk (**a**, **b**, **e**) and profitability risk (**c**, **d**, **f**), and future projected SST dynamics (**g**). Panels (**a**) to (**d**) show risk dynamics for the individual management policies; panels (**e**) and (**f**) show dynamics of median (solid line) and 25- and 75-percentile risk over policies. Colors in (**e**) and (**f**) represent the exploitation metric: orange: constant catch, purple: constant harvest rate. Black line in (**g**) represents mean annual projected SST in the North Sea over RCP scenarios 4.5 and 8.5; colors represent projections for the single scenarios (orange: RCP4.5, purple: RCP8.5).

Temporal trends in risk increase matched the increasing trend observed in projected future SST dynamics in the two RCP scenarios (Fig. 5), especially in the constant-harvest-rate policies: The period of stronger SST increase starting in the 2060s corresponds to more marked increases in sustainability risk (median over all policies: c. 30% in 2060 to c. 45% in 2100) and profitability risk (c. 50% in 2030 to c. 60% in 2100) than before (Fig. 5e, f). Risk variability over time was relatively small compared to risk variability over policies, however. Notably, even at low fishing levels (catch < 50 kt; harvest rate < 15%), risk increased strongly from rather low levels (< < 25%) after only few (appx. five) years (Fig. 5a, b). Aggregated risks for constant-catch policies were overall less variable over time than risks for constant-harvest-rate policies but also much higher in magnitude (median over all policies never < 75% after 2035); lower catch levels appeared to result in a stronger temporal sustainability-risk signal largely matching that obtained from constant-harvest-rate policies (Fig. 5a, b; see also Supplementary Results 2). Furthermore, risk increase was relatively steady under lower catch levels (< 100 kt) and over most of the range of harvest-rate policies, but regularly peaked under high-catch policies (> 100 kt) (Fig. 5a, b), a pattern likely related to density dependence in the SR relationship (see Supplementary Fig. 4, Supplementary Results 3).

Profitability risk increased over time following a similar trend as the increase in sustainability risk, especially under the harvest-rate policies associated with lower risk (Fig. 5a, c, f); very low levels of risk (< < 25%) were only achieved under rather high fishing levels (> 50 kt catch, > 20% harvest rate) and only for a brief initial period (the first appx. 2–3 years).

## Discussion

We here developed a novel approach to evaluate management strategies for commercially exploited fish stocks that unlike traditional application followed Robust Decision Making (RDM) protocols. RDM shifts emphasis from improving model predictions through increasing model complexity to improving management decisions^25^. RDM hence seeks to increase the understanding about the consequences of management actions under a large spectrum of possible scenarios, eventually defining a management strategy that is robust to a multitude of equally possible futures^36^. Our RDM projection study, applied to North Sea cod, consequently inverted the notion of poor predictability of stock dynamics limiting climate-informed advice^47^ into an explorative, policies-oriented evaluation of the potential to achieve sustainable management of this depleted fish stock given uncertainties in the recruitment process and the future course of climate change.

A major result of our study is that uncertainty about future recruitment under climate change has a similar impact on management success as the harvest control rule strategies we applied. Uncertainty in recruitment is a well-known challenge for biomass projections and specification of harvest levels for exploited fish stocks^8,84^. Our study goes beyond this general knowledge and demonstrates that density-independent productivity of the stock and the strength of the negative effect of increasing SSTs (reflected by the *log(alpha)* and *gamma* parameters in a SR model, respectively) are of predominant importance for management success in our simulations of North Sea cod. The importance of *log(alpha)* points towards the long-standing discussion in fisheries science whether compensatory or depensatory (i.e. the Allee effect) processes dominate at low stock sizes^85^. If depensation prevails, recovery of overexploited stocks is inhibited and has been shown to exist especially for cod populations^86–89^ and recently for North Sea cod^90^. Empirical evidence is however overall stronger for compensatory effects in fish stocks, i.e. increasing productivity at low stock sizes and hence high recovery potential^85^. Nevertheless, our results reinforce that critically low stock sizes should be avoided to not critically endanger fish stocks and to not impede their recovery when depleted^44,91–93^.

Our study reinforces that climate change is challenging fisheries management because it introduces further sources of uncertainty to the decision-making process^94–98^. We focused on evaluating the importance of uncertainty in recruitment, because it is likely the most important process affected by the consequences of climate change in the ocean^99^ especially in North Sea cod^100–102^. Nevertheless, our model remains a gross simplification of the many climate-related processes, including in addition to SST also e.g. plankton abundance^102^, that affect not only the recruitment of cod in the North Sea, but also growth^103^ and distribution shifts^104^. Furthermore, finding relationships between environmental variables and recruitment is difficult because these notoriously have a poor fit^105^. The importance of uncertainty in the *gamma* parameter (reflecting the strength of the negative effect of increasing SSTs) for sustainable management in our simulations demonstrates the vulnerability of management approaches that consider only low-level uncertainty in the climate effect on recruitment. Moreover, uncertainty in SR model parameterization was more important than the type of emission scenario, revealing that considering the future course of climate change is less decisive than structural uncertainty in the model. Nevertheless, matching dynamics of risk and SST increase towards the end of the century indicate that the degree of future warming will still likely have a considerable impact on North Sea cod productivity. This is also reflected in significantly reduced recruitment and SSB at RCP8.5 compared to RCP4.5 in that period (Supplementary Fig. 4). In light of the massive effect of SR uncertainty found in our study, it would be worthwhile to apply our RDM approach to an extended range of SR functions and environmental and biological covariates in future studies, especially for potential operational management applications. Further, an operational RDM application should consider the impact of DU given the present state of the stock to inform short-term management decisions, in addition to scenario simulations initialized with the assumption of a rebuilt stock as presented here.

A further major result of our study is that none of the management strategies we applied in our simulations is fully robust to the uncertainty in model parameterization and future climate change. Specifically, no constant catch or harvest rate was able to meet sustainability targets for North Sea cod over the entire simulation period; even at low fishing levels, risk increased from low levels only few years into the future. However, a harvest-rate strategy provided a safer operation space with a threshold-like transition to less safe exploitation levels than a constant catch strategy with its less-distinctly bounded space. For the latter, it was only possible to determine a policy range less affected by high-risk periods (and therefore likely less affected by temporal recruitment variability), but no distinct low-risk policy range. These results confirms the theory that while providing stable catches, a constant catch strategy may lead to excessive exploitation rates at low stock sizes, while a constant-F strategy is more responsive to fluctuations in stock size^52,53^. Our harvest rate strategy corresponds effectively to a constant F strategy^54^. However, because we were not primarily interested in finding the better management strategy, but rather exploring the effect of uncertainties on successful management, we used harvest rate, and considered F_MSY_, in addition to MSY B_trigger_, as one of our management targets under both harvest control rules.

Using both a target F and a limit biomass reference point, we mimicked the MSY strategy implemented in EU fisheries management by ICES^106,107^. We however disregarded the threshold F rule implemented which is likely the most resilient management approach to uncertainties and climate change effects^54,108,109^, but was not useful to implement in our study, as some unfavourable scenarios might have enforced a permanent down-scaling of F and thus reduced the validity of results attributed to certain harvesting levels (especially where sustainability was achieved with the permanently reduced F). Stress-testing the EU MSY strategy under climate change scenarios would hence be a valuable study.

Our approach employed the official F and biomass reference points^41^ that are based on ICES’ assumption of a hockey-stick SR relationship without environmental covariates^68^. Management reference points are regularly updated in the so-called ICES benchmark process^75^, in response to productivity changes in the stock (or changes to productivity perception) founded in a changed (or differently perceived) SR relationship. We, however, did not adapt reference points to the various SR relationships utilized in our projections, as we do not assume that future recruitment will follow any of these relationships to a reliable degree. Rather, projecting with the large variety of SR relationships here represents an expression of the inability of predicting recruitment reliably, and the calculation of SR-specific reference points (and evaluation of projected SSB and F against them) would not be meaningful in this context. Also, the effectiveness of flexible reference points in general is historically questionable^74^ and in simulations strongly depends on limited uncertainty^96,110^, and can even result in poorer management outcome^76,98^^.^ We hence adopted a conservationist perspective and consider MSY B_trigger_ as the lower limit to good stock status, and F_MSY_ as the upper limit to ecologically acceptable fishing pressure, and evaluated projected SSB and F against them to assess policy performance under deep uncertainty in predicting recruitment.

Given that our simulations for North Sea cod revealed no management strategy that is fully robust to uncertainty in model parameterization and future climate change, we conducted a risk and trade-off analysis, exploring the trade-off between the risk of not achieving sustainability targets and the risk of the fishery of not operating in a profitable way. Such a risk assessment can be valuable decision support tool for fisheries managers that usually must consider both ecological and economic (and hence social) objectives. For North Sea cod our results indicate that even the best trade-offs of sustainability and profitability would require low catches or harvest rates compared to historical levels, reflecting the presently low productivity of the stock as integrated in the deep uncertainty about the SR relationship. Indeed, the rather immediate over-fishing associated with early attainment of very low profitability risk (which was associated with high fishing levels and which rapidly and markedly increased) implies that such low fishing levels are required even in the short-term where the impact of DU is still reduced. In the mid-to-long term, however, even low fishing levels would not be sufficient to fully compensate for DU effects and for the impacts of stronger warming on productivity, as reflected both by considerable sustainability- and profitability risks. Our profitability reference level was set quite arbitrary to a mean over years 2000 to 2018, and hence further sensitivity studies would be required for an extended use. Our representation of the economy in our modelling approach is furthermore quite simplistic since North Sea cod is usually caught in a mixed fishery^41^ that would affect the profitability of the respective fleets^111^. We are nevertheless convinced that this first approximation of profitability holds for our single-species approach.

An additional constraint to direct practical implementation, our approach deviates from formal management strategy evaluation (MSE) in fisheries science by not simulating observation- and implementation errors, and not simulating future stock assessments and reference-point re-estimations (as outlined in e.g.^23^), as we adopted a more theoretical approach focusing on the impact of deep uncertainties on long-term policy success. We suggest our approach as a pre-analysis to classical MSE (i.e., a form of sensitivity analysis concerning recruitment uncertainties). Extended studies could furthermore aim at an integration into the existing/more applied MSE model frameworks.

Further complications arise from the population structure of North Sea cod, which is comprised of three geographically distinct sub-populations with different life-history traits and productivity levels (summarized by^112^) and which are recognized in management since recently^113,114^. We selected the former one-stock formulation^41^ in order to maintain a relatively simple model structure with a correspondingly limited number of uncertainties to illustrate the RDM approach, but suggest an update to the current stock perception (and future updates in case of any future changes to stock structure or stock perception) for a potential operational application.

Finally, dynamic changes unrelated to climate change in the North Sea also have the potential to affect future productivity of North Sea cod: For example, offshore windfarms in the southern North Sea provide novel habitat for demersal/rock-associated species, there are indications of their usage as spawning grounds by cod^115,116^. With construction of windfarms is expected to increase in the North Sea in the future, population-level impacts on North Sea cod might hypothetically occur in the future, however research on the subject has not yet progressed to a point where such an impact could be included in a population model.

In conclusion, we here provided the first study that considered principles of decision-making under deep uncertainties (DMDU) in a fisheries management framework. Our study contributes a novel aspect to MSE approaches in fisheries by taking the principle to consider multiple operating models with multiple assumptions about the impact of climate change^23,117^ to its extremes, thereby accounting for uncertainty in stock productivity in a more holistic way. We furthermore show how robust decision-making (RDM) approaches can support a management system to consider and to cope with deep uncertainties by considering risks and trade-offs between multiple goals. Arguably, our single-species approach is simplistic compared to state-of-the-art multispecies or food web modelling approaches^97,118^, but allowed us to follow the RDM philosophy of shifting emphasis from improving model predictions to improving management decisions^25^. We consider our approach as an addition to the toolbox in ecosystem-based fisheries management approaches that are instrumental in developing a sustainable exploitation of our world fisheries resources.

### Supplementary Information

Supplementary Information.

## Data Availability

Model input data and code are available on https://github.com/imf-uham/DMDU_North_Sea/. Model output data are available on https://zenodo.org/records/11110075.

## References

[1] Lotze, *et al.* Global ensemble projections reveal trophic amplification of ocean biomass declines with climate change. *PNAS* **116**, 12907–12912. 10.1073/pnas.1900194116 (2019). 31186360 10.1073/pnas.1900194116 PMC6600926

[2] Tittensor, D. P. *et al.* Next-generation ensemble projections reveal higher climate risks for marine ecosystems. *Nat. Clim. Change* **11**, 973–981. 10.1038/s41558-021-01173-9 (2021). 10.1038/s41558-021-01173-9 PMC8556156 34745348

[3] Haltuch, M. A. *et al.* Unraveling the recruitment problem: A review of environmentally-informed forecasting and management strategy evaluation. *Fish. Res.* **217**, 198–216. 10.1016/j.fishres.2018.12.016 (2019). 10.1016/j.fishres.2018.12.016

[4] Hill, S. L. *et al.* Model uncertainty in the ecosystem approach to fisheries. *Fish Fish.* **8**, 315–336. 10.1111/j.1467-2979.2007.00257.x (2007). 10.1111/j.1467-2979.2007.00257.x

[5] Payne, M. R. *et al.* Uncertainties in projecting climate-change impacts in marine ecosystems. *ICES J. Mar. Sci.* **73**, 1272–1282. 10.1093/icesjms/fsv231 (2016). 10.1093/icesjms/fsv231

[6] Szuwalski, C. S. & Hollowed, A. B. Climate change and non-stationary population processes in fisheries management. *ICES J. Mar. Sci.* **73**, 1297–1305. 10.1093/icesjms/fsv229 (2016). 10.1093/icesjms/fsv229

[7] Pineda, J., Reyns, N. B. & Starczak, V. R. Complexity and simplification in understanding recruitment in benthic populations. *Popul. Ecol.* **51**, 17–32. 10.1007/s10144-008-0118-0 (2009). 10.1007/s10144-008-0118-0

[8] Collie, J. S., Bell, R. J., Collie, S. B. & Minto, C. Harvest strategies for climate-resilient fisheries. *ICES J. Mar. Sci.* **8**, 2774–2783. 10.1093/icesjms/fsab152 (2021). 10.1093/icesjms/fsab152

[9] Houde, E. D. Fish early life dynamics and recruitment variability. *Am. Fish. Soc. Symp.* **2**, 17–29 (1987).

[10] Lomartire, S., Marques, J. C. & Gonçalves, A. M. M. The key role of zooplankton in ecosystem services: A perspective of interaction between zooplankton and fish recruitment. *Ecol. Indic.* **129**, 107867. 10.1016/j.ecolind.2021.107867 (2021). 10.1016/j.ecolind.2021.107867

[11] Nilssen, E. M., Pedersen, T., Hopkins, C. C. E., Thyholt, K. & Pope, J. G. Recruitment variability and growth of Northeast arctic cod: Influence of physical environment, demography and predator-prey energetics. *ICES Mar. Sci. Symp.* **198**, 449–470 (1994).

[12] Macura, B. *et al.* Impact of structural habitat modifications in coastal temperate systems on fish recruitment: A systematic review. *Environ. Evid.* **8**, 14. 10.1186/s13750-019-0157-3 (2019). 10.1186/s13750-019-0157-3

[13] Tiedemann, M., Slotte, A., Nash, R. D. M., Stenevik, E. K. & Kjesbu, O. S. Drift Indices confirm that rapid larval displacement is essential for recruitment success in high-latitude oceans. *Front. Mar. Sci.* **8**, 679900. 10.3389/fmars.2021.679900 (2021). 10.3389/fmars.2021.679900

[14] Myers, R. A. & Barrowman, N. J. Is fish recruitment related to spawner abundance?. *Fish. Bull.* **94**, 707–724 (1996).

[15] Szuwalski, C. S. *et al.* Global forage fish recruitment dynamics: A comparison of methods, time-variation, and reverse causality. *Fish. Res.* **214**, 56–64. 10.1016/j.fishres.2019.01.007 (2019). 10.1016/j.fishres.2019.01.007

[16] Basson, M. The importance of environmental factors in the design of management procedures. *ICES J. Mar. Sci.* **56**, 933–942. 10.1006/jmsc.1999.0541 (1999). 10.1006/jmsc.1999.0541

[17] Walker, W. E., Lempert, R. J. & Kwakkel, J. H. Deep Uncertainty. In *Encyclopedia of Operations Research and Management Science* (eds Gass, S. I. & Fu, M. C.) 395–402 (Springer US, 2013). 10.1007/978-1-4419-1153-7_1140.

[18] Courtney, H. *20/20 Foresight: Crafting Strategy in an Uncertain World* 209 (Harvard Business School Press, 2001).

[19] Walker, W. E. *et al.* Defining uncertainty: A conceptual basis for uncertainty management in model-based decision support. *Integr. Ass.* **4**, 5–7. 10.1076/iaij.4.1.5.16466 (2003). 10.1076/iaij.4.1.5.16466

[20] Marchau, V. A. W. J., Walker, W. E., Bloemen, P. J. T. M. & Popper, S. W. Introduction. In *Decision Making under Deep Uncertainty: From Theory to Practice* (eds Marchau, V. A. W. J. *et al.*) 1–20 (Springer International Publishing, 2019). 10.1007/978-3-030-05252-2_1.

[21] Bloemen, P. J. T. M., Hammer, F., van der Vlist, M. J., Grinwis, P. & van Alphen, J. DMDU into Practice: Adaptive Delta Management in the Netherlands. In *Decision Making under Deep Uncertainty: From Theory to Practice* (eds Marchau, V. A. W. J. *et al.*) 321–351 (Springer International Publishing, 2019). 10.1007/978-3-030-05252-2_14.

[22] Vaghefi, S. A., Muccione, V., van Ginkel, K. C. H. & Haasnoot, M. Using decision making under deep uncertainty (DMDU) approaches to support climate change adaptation of Swiss Ski resorts. *Environ. Sci. Policy* **126**, 65–78. 10.1016/j.envsci.2021.09.005 (2021). 10.1016/j.envsci.2021.09.005

[23] Punt, A. E., Butterworth, D. S., de Moor, C. L., de Oliveira, J. A. A. & Haddon, M. Management strategy evaluation: Best practices. *Fish Fish.* **17**, 303–334. 10.1111/faf.12104 (2016). 10.1111/faf.12104

[24] Blamey, L. K. *et al.* Redesigning harvest strategies for sustainable fishery management in the face of extreme environmental variability. *Conserv. Biol.* **36**, 13864. 10.1111/cobi.13864 (2021). 10.1111/cobi.13864 34929068

[25] Lempert, R. J. Robust Decision Making (RDM). In *Decision Making under Deep Uncertainty: From Theory to Practice* (eds Marchau, V. A. W. J. *et al.*) 23–51 (Springer International Publishing, 2019). 10.1007/978-3-030-05252-2_2.

[26] Rochet, M.-J. & Rice, J. C. Simulation-based management strategy evaluation: Ignorance disguised as mathematics?. *ICES J. Mar. Sci.* **66**, 754–762. 10.1093/icesjms/fsp023 (2009). 10.1093/icesjms/fsp023

[27] Lempert, R. J., Nakicenovic, N., Sarewitz, D. & Schlesinger, M. Characterizing climate-change uncertainties for decision-makers. An editorial essay. *Clim. Change* **65**, 1–9. 10.1023/B:CLIM.0000037561.75281.b3 (2004). 10.1023/B:CLIM.0000037561.75281.b3

[28] Howell, D., Filin, A. A., Bogstad, B. & Stiansen, J. E. Unquantifiable uncertainty in projecting stock response to climate change: Example from North East Arctic cod. *Mar. Biol. Res.* **9**, 920–931. 10.1080/17451000.2013.775452 (2013). 10.1080/17451000.2013.775452

[29] Schindler, D. E. & Hilborn, R. Prediction, precaution, and policy under global change. *Science* **347**, 953–954. 10.1126/science.1261824 (2015). 25722401 10.1126/science.1261824

[30] Lempert, R., Popper, S. & Bankes, S. *Shaping the Next One Hundred Years: New Methods for Quantitative, Long-Term Policy Analysis* (RAND Corporation, 2003). 10.7249/MR1626.

[31] Lempert, R. J. *et al.**Making Good Decisions Without Predictions: Robust Decision Making for Planning Under Deep Uncertainty* 6 (RAND Corporation, 2013). 10.7249/RB9701.

[32] Walker, W. E., Rahman, S. A. & Cave, J. Adaptive policies, policy analysis, and policy-making. *Eur. J. Oper. Res.* **128**, 282–289. 10.1016/S0377-2217(00)00071-0 (2001). 10.1016/S0377-2217(00)00071-0

[33] Walker, W. E., Marchau, V. A. W. J. & Kwakkel, J. H. Dynamic Adaptive Planning (DAP). In *Decision Making under Deep Uncertainty: From Theory to Practice* (eds Marchau, V. A. W. J. *et al.*) 53–69 (Springer International Publishing, 2019). 10.1007/978-3-030-05252-2_3.

[34] Haasnoot, M., Kwakkel, J. H., Walker, W. E. & ter Maat, J. Dynamic adaptive policy pathways: A method for crafting robust decisions for a deeply uncertain world. *Glob. Environ. Change* **23**, 485–498. 10.1016/j.gloenvcha.2012.12.006 (2013). 10.1016/j.gloenvcha.2012.12.006

[35] Pielke, R. A. Jr., Sarewitz, D. & Byerly, R. Jr. Decision Making and the Future of Nature: Understanding and Using Predictions. In *Prediction Science, Decision Making, and the Future of Nature* (eds Sarewitz, D. *et al.*) 361–387 (Island Press, 2000).

[36] Lempert, R. J. & Popper, S. W. High-Performance Government in an Uncertain World. In *High-Performance Government: Structure, Leadership, Incentives* (eds Klitgaard, R. & Light, P. C.) 113–136 (RAND Corporation, 2005).

[37] Hadjimichael, A., Reed, P. M. & Quinn, J. D. Navigating deeply uncertain tradeoffs in harvested predator-prey systems. *Complexity* **2020**, 1–18. 10.1155/2020/4170453 (2020). 10.1155/2020/4170453

[38] Wainger, L. A. *et al.* (2021) Decision Making under Deep Uncertainty—What is it and how might NOAA use it? Report to the Science Advisory Board from the Ecosystem Science and Management Working Group. NOAA, Washington, D.C. 16

[39] Villasante, S., Rodríguez-Gónzalez, D. & Antelo, M. On the non-compliance in the North Sea cod stock. *Sustainability* **5**, 1974–1993. 10.3390/su5051974 (2013). 10.3390/su5051974

[40] Blanchard, J. L., Heffernan, O. A. and Fox, C. J. North Sea (ICES Divisions IVa-c and VIId). in *ICES Cooperative Research Report No. 274: Spawning and life history information for North Atlantic cod stocks*, (Brander, K.) 76–88 (ICES, 2005); 10.17895/ices.pub.5478

[41] ICES. Cod (*Gadus morhua*) in Subarea 4, Division 7.d, and Subdivision 20 (North Sea, eastern English Channel, Skagerrak). ICES Working Group on the Assessments of Demersal Stocks in the North Sea and Skagerrak, **3 (66),** 79–162; 10.17895/ices.pub.8211 (2021).

[42] Rose, G. A., Marteinsdottír, G. & Godø, O.-R. Exploitation: Cod is Fish and Fish is Cod. In *Atlantic Cod: A Bio-Ecology* (ed. Rose, G. A.) 287–336 (Wiley, 2019). 10.1002/9781119460701.ch7.

[43] Hutchings, J. A. & Reynolds, J. D. Marine fish population collapses: Consequences for recovery and extinction risk. *BioScience* **54**, 297–309. 10.1641/0006-3568(2004)054[0297:MFPCCF]2.0.CO;2 (2004). 10.1641/0006-3568(2004)054[0297:MFPCCF]2.0.CO;2

[44] Sguotti, C. *et al.* Catastrophic dynamics limit Atlantic cod recovery. *Proc. R. Soc. B* **286**, 20182877. 10.1098/rspb.2018.2877 (2019). 30862289 10.1098/rspb.2018.2877 PMC6458326

[45] Sguotti, C. *et al.* Non-linearity in stock–recruitment relationships of Atlantic cod: Insights from a multi-model approach. *ICES J. Mar. Sci.* **77**, 1492–1502. 10.1093/icesjms/fsz113 (2020). 10.1093/icesjms/fsz113

[46] Blöcker, A. M. *et al.* Regime shift dynamics, tipping points and the success of fisheries management. *Sci. Rep.* **13**, 289. 10.1038/s41598-022-27104-y (2023). 36609587 10.1038/s41598-022-27104-y PMC9822959

[47] Planque, B., Fox, C. J., Saunders, M. A. & Rockett, P. On the prediction of short term changes in the recruitment of North Sea cod (*Gadus morhua*) using statistical temperature forecasts. *Sci. Mar.* **67**, 211–218. 10.3989/scimar.2003.67s1211 (2003). 10.3989/scimar.2003.67s1211

[48] Sguotti, C. *et al.* Stable landings mask irreversible community reorganizations in an overexploited Mediterranean ecosystem. *J. Anim. Ecol.* **91**, 2465–2479. 10.1111/1365-2656.13831 (2022). 36415049 10.1111/1365-2656.13831

[49] Sguotti, C., Färber, L. & Romagnoni, G. Regime Shifts in Coastal Marine Ecosystems: Theory, Methods and Management Perspectives. In *Reference Module in Earth Systems and Environmental Sciences* (ed. Sguotti, C.) (Elsevier BV, 2022). 10.1016/B978-0-323-90798-9.00004-4.

[50] NRC *Informing Decisions in a Changing Climate*. 200. (The National Academy Press, 2009) 10.17226/12626.

[51] Walters, C. J. & Martell, S. J. D. *Fisheries Ecology and Management* 448 (Princeton University Press, 2005).

[52] Deroba, J. J. & Bence, J. R. A review of harvest policies: Understanding relative performance of control rules. *Fish. Res.* **94**, 210–223. 10.1016/j.fishres.2008.01.003 (2008). 10.1016/j.fishres.2008.01.003

[53] Restrepo, V. R. & Powers, J. E. Precautionary control rules in US fisheries management: Specification and performance. *ICES J. Mar. Sci.* **56**, 846–852. 10.1006/jmsc.1999.0546 (1999). 10.1006/jmsc.1999.0546

[54] Free, C. M. *et al.* Harvest control rules used in US federal fisheries management and implications for climate resilience. *Fish Fish.* **24**, 248–262. 10.1111/faf.12724 (2022). 10.1111/faf.12724

[55] Allen, R. L. Models for fish populations: A review. *New Zeal. Oper. Res.* **4**, 1–20 (1975).

[56] Serpetti, N. *et al.* Impact of ocean warming on sustainable fisheries management informs the ecosystem approach to fisheries. *Sci. Rep.* **7**, 13438. 10.1038/s41598-017-13220-7 (2017). 29044134 10.1038/s41598-017-13220-7 PMC5647405

[57] Subbey, S., Devine, J. A., Schaarschmidt, U. & Nash, R. D. M. Modelling and forecasting stock-recruitment: Current and future perspectives. *ICES J. Mar. Sci.* **71**, 2307–2322. 10.1093/icesjms/fsu148 (2014). 10.1093/icesjms/fsu148

[58] Schenk, H., Zimmermann, F. & Quaas, M. The economics of reversing fisheries-induced evolution. *Nat. Sustain.* **6**, 706–711. 10.1038/s41893-023-01078-9 (2023). 10.1038/s41893-023-01078-9

[59] Huang, B. *et al.* Extended reconstructed sea surface temperature, Version 5 (ERSSTv5): Upgrades, Validations, and Intercomparisons. *J. Clim.* **30**, 8179–8205. 10.1175/JCLI-D-16-0836.1 (2017). 10.1175/JCLI-D-16-0836.1

[60] Peck, M. A. *et al.**Climate Change and European Fisheries and Aquaculture CERES Project Synthesis Report* 110 (Universität Hamburg, 2020). 10.25592/uhhfdm.804.

[61] Maraun, D. Bias correcting climate change simulations—a critical review. *Curr. Clim. Change Rep.* **2**, 211–220. 10.1007/s40641-016-0050-x (2016). 10.1007/s40641-016-0050-x

[62] BLE. Monatsbericht 2020. Bericht über die Fischerei und die Marktsituation für Fischereierzeugnisse in der Bundesrepublik Deutschland. 49. (German federal office for agriculture and food [BLE], 2020)

[63] Ricker, W. E. Stock and recruitment. *J. Fish. Res. Board Can.* **11**, 559–623. 10.1139/f54-039 (1954). 10.1139/f54-039

[64] Beverton, R. J. H. & Holt, S. J. *On the Dynamics of Exploited Fish Populations* (Chapman & Hall, 1957).

[65] Ricker, W. E. Computation and interpretation of biological statistics of fish populations. *Bull. Fish. Res. Board Can.*10.2307/3800109 (1975). 10.2307/3800109

[66] Hilborn, R. & Walters, C. J. *Quantitative Fisheries Stock Assessment. Choice, Dynamics and Uncertainty* 570 (Chapman and Hall, 1992). 10.1007/978-1-4615-3598-0.

[67] Patterson, K. *et al.* Estimating uncertainty in fish stock assessment and forecasting. *Fish Fish.* **2**, 125–157. 10.1046/j.1467-2960.2001.00042.x (2001). 10.1046/j.1467-2960.2001.00042.x

[68] ICES. Cod (27.47d20) Benchmark workshop on North sea stocks (WKNSEA). *ICES Scientific Reports* **3**(25), 5–46. 10.17895/ices.pub.7922 (2021). 10.17895/ices.pub.7922

[69] van Vuuren, D. P. *et al.* The representative concentration pathways: An overview. *Clim. Change* **109**, 5–31. 10.1007/s10584-011-0148-z (2011). 10.1007/s10584-011-0148-z

[70] Moss, R. H. *et al.* The next generation of scenarios for climate change research and assessment. *Nature* **463**, 747–756. 10.1038/nature088234 (2010). 20148028 10.1038/nature088234

[71] ICES. ICES Advice basis in *report of the ICES advisory committee, 2019, ICES Advice 2019, Introduction_to_advice_2019*. 17. (ICES, 2019); 10.17895/ices.advice.5757

[72] ICES. ICES fisheries reference points for category 1 and 2 stocks; Technical Guidelines in *Report of the ICES Advisory Committee, 2021. ICES Advice 2021, Section 16.4.3.1*. 19 (ICES, 2021); 10.17895/ices.advice.7891.

[73] Mace, P. M. A new role for MSY in single-species and ecosystem approaches to fisheries stock assessment and management. *Fish Fish.* **2**, 2–32. 10.1046/j.1467-2979.2001.00033.x (2001). 10.1046/j.1467-2979.2001.00033.x

[74] Silvar-Viladomiu, P. *et al.* Moving reference point goalposts and implications for fisheries sustainability. *Fish Fish.* **22**, 1345–1358. 10.1111/faf.12591 (2021). 10.1111/faf.12591

[75] ICES. ICES Guidelines for Benchmarks. Version 1. ICES Guidelines and Policies—Advice Technical Guidelines. 26 10.17895/ices.pub.22316743

[76] Travers-Trolet, M., Bourdaud, P., Genu, M., Velez, L. & Vermard, Y. The risky decrease of fishing reference points under climate change. *Front. Mar. Sci.* **7**, 568232. 10.3389/fmars.2020.568232 (2020). 10.3389/fmars.2020.568232

[77] Friedman, J. H. Greedy function approximation: A gradient boosting machine. *Ann. Stat.* **29**, 1189–1232. 10.1214/aos/1013203451 (2001). 10.1214/aos/1013203451

[78] van Rossum, G. *Python Tutorial Technical Report CS R9526* 71 (Centrum voor Wiskunde en Informatica (CWI), 1995).

[79] Kwakkel, J. H. The exploratory modeling workbench: An open source toolkit for exploratory modeling, scenario discovery, and (multi-objective) robust decision making. *Environ. Model. Softw.* **96**, 239–250. 10.1016/j.envsoft.2017.06.054 (2017). 10.1016/j.envsoft.2017.06.054

[80] Pedregosa, F. *et al.* Scikit learn: Machine learning in python. *J. Mach. Learn. Res.* **12**, 2825–2830 (2011).

[81] R Core Team R: an environment for statistical computing. R Foundation for Statistical Computing, Vienna. URL https://www.R-project.org/. (2020) Last access on 15^th^ June, 2023

[82] Wickham, H. *ggplot2: Elegant graphics for data analysis* 213 (Springer, 2016).

[83] Hunter, J. D. Matplotlib: A 2D graphics environment. *Comput. Sci. Eng.* **9**, 90–95. 10.1109/MCSE.2007.55 (2007). 10.1109/MCSE.2007.55

[84] Wiedenmann, J. & Jensen, O. P. Uncertainty in stock assessment estimates for New England groundfish and its impact on achieving target harvest rates. *Can. J. Fish. Aquat. Sci.* **75**, 342–356. 10.1139/cjfas-2016-0484 (2017). 10.1139/cjfas-2016-0484

[85] Hilborn, R., Hively, D. J., Jensen, O. P. & Branch, T. A. The dynamics of fish populations at low abundance and prospects for rebuilding and recovery. *ICES J. Mar. Sci.* **71**, 2141–2151. 10.1093/icesjms/fsu035 (2014). 10.1093/icesjms/fsu035

[86] Rowe, S., Hutchings, J. A., Bekkevold, D. & Rakitin, A. Depensation, probability of fertilization, and the mating system of Atlantic cod (*Gadus**morhua* L.). *ICES J. Mar. Sci.* **61**, 1144–1150. 10.1016/j.icesjms.2004.07.007 (2004). 10.1016/j.icesjms.2004.07.007

[87] Keith, D. M. & Hutchings, J. A. Population dynamics of marine fishes at low abundance. *Can. J. Fish. Aquat. Sci.* **69**, 1150–1163. 10.1139/F2012-055 (2012). 10.1139/F2012-055

[88] Kuparinen, A., Keith, D. M. & Hutchings, J. A. Allee effects and the uncertainty of population recovery. *Conserv. Biol.* **28**, 790–798. 10.1111/cobi.12216 (2014). 24512300 10.1111/cobi.12216

[89] Neuenhoff, R. D. *et al.* Continued decline of a collapsed population of Atlantic cod (*Gadus morhua*) due to predation-driven Allee effects. *Can. J. Fish. Aquat. Sci.* **76**, 168–184. 10.1139/cjfas-2017-0190 (2018). 10.1139/cjfas-2017-0190

[90] Winter, A.-M., Richter, A. & Eikeset, A. M. Implications of Allee effects for fisheries management in a changing climate: Evidence from Atlantic cod. *Ecol. Appl.* **30**, e01994. 10.1002/eap.1994 (2019). 31468660 10.1002/eap.1994

[91] Britten, G. L., Dowd, M., Kanary, L. & Worm, B. Extended fisheries recovery timelines in a changing environment. *Nat. Commun.* **8**, 15325. 10.1038/ncomms15325 (2017). 28524851 10.1038/ncomms15325 PMC5493592

[92] Gaines, S. D. *et al.* Improved fisheries management could offset many negative effects of climate change. *Sci. Adv.* **4**, eaao1378 (2018). 30167455 10.1126/sciadv.aao1378 PMC6114984

[93] Möllmann, C. *et al.* Tipping point realized in cod fishery. *Sci. Rep.* **11**, 14259. 10.1038/s41598-021-93843-z (2021). 34253825 10.1038/s41598-021-93843-z PMC8275682

[94] Brander, K. M. Global fish production and climate change. *PNAS* **104**, 19709–19714. 10.1073/pnas.0702059104 (2007). 18077405 10.1073/pnas.0702059104 PMC2148362

[95] Miller, K. *et al.* Climate change, uncertainty, and resilient fisheries: Institutional responses through integrative science. *Progr. Oceanogr.* **87**, 338–346. 10.1016/j.pocean.2010.09.014 (2010). 10.1016/j.pocean.2010.09.014

[96] Punt, A. E. *et al.* Fisheries management under climate and environmental uncertainty: control rules and performance simulation. *ICES J. Mar. Sci.* **71**, 2208–2220. 10.1093/icesjms/fst057 (2014). 10.1093/icesjms/fst057

[97] Holsman, K. K. *et al.* Ecosystem-based fisheries management forestalls climate-driven collapse. *Nat. Commun.* **11**, 4579. 10.1038/s41467-020-18300-3 (2019). 10.1038/s41467-020-18300-3 PMC7486947 32917860

[98] Szuwalski, C. S. *et al.* Unintended consequences of climate-adaptive fisheries management targets. *Fish Fish.* **24**, 439–453. 10.1111/faf.12737 (2023). 10.1111/faf.12737

[99] Britten, G. L., Dowd, M. & Worm, B. Changing recruitment capacity in global fish stocks. *PNAS* **113**, 134–139. 10.1073/pnas.150470911 (2015). 26668368 10.1073/pnas.150470911 PMC4711852

[100] O’Brien, C. M., Fox, C. J., Planque, B. & Casey, J. Climate variability and North Sea cod. *Nature* **404**, 142. 10.1038/35004654 (2000). 10724155 10.1038/35004654

[101] Beaugrand, G., Brander, K. M., Lindley, J. A., Souissi, S. & Reid, P. C. Plankton effect on cod recruitment in the North Sea. *Nature* **426**, 661–664. 10.1038/nature02164 (2003). 14668864 10.1038/nature02164

[102] Olsen, E. M. *et al.* Spawning stock and recruitment in North Sea cod shaped by food and climate. *Proc. R. Soc. B* **278**, 504–510. 10.1098/rspb.2010.1465 (2011). 20810442 10.1098/rspb.2010.1465 PMC3025682

[103] Pilling, G. M., Millner, R. S., Easey, M. W., Maxwell, D. L. & Tidd, A. N. Phenology and North Sea cod *Gadus**morhua* L.: has climate change affected otolith annulus formation and growth?. *J. Fish Biol.* **70**, 584–599. 10.1111/j.1095-8649.2007.01331.x (2007). 10.1111/j.1095-8649.2007.01331.x

[104] Engelhard, G. H., Righton, D. A. & Pinnegar, J. K. Climate change and fishing: a century of shifting distribution in North Sea cod. *Glob. Change Biol.* **20**, 2473–2484. 10.1111/gcb.12513 (2013). 10.1111/gcb.12513 PMC4282283 24375860

[105] Myers, R. A. When do environment-recruitment correlations work?. *Rev. Fish. Biol. Fish.* **8**, 285–305. 10.1023/A:1008828730759 (1998). 10.1023/A:1008828730759

[106] European Union Regulation (EU) No 1380/2013 of the European Parliament and of the Council of 11 December 2013 on the common fisheries policy, amending council regulations (EC) No 1954/2003 and (EC) No 1224/2009 and repealing Council Regulations (EC) No 2371/2002 and (EC) No 639/2004 and Council Decision 2004/585/EC. *OJEU*, **L 354**, 22–61 (2013)

[107] ICES. General context of ICES advice. *ICES Advice: Recurrent Advice. Report.*10.17895/ices.advice.18667652.v1 (2012). 10.17895/ices.advice.18667652.v1

[108] Kritzer, J. P., Costello, C., Mangin, T. & Smith, S. L. Responsive harvest control rules provide inherent resilience to adverse effects of climate change and scientific uncertainty. *ICES J. Mar. Sci.* **76**, 1424–1435. 10.1093/icesjms/fsz038 (2019). 10.1093/icesjms/fsz038

[109] Mildenberger, T. K. *et al.* Implementing the precautionary approach into fisheries management: Biomass reference points and uncertainty buffers. *Fish Fish* **23**, 73–92. 10.1111/faf.12599 (2021). 10.1111/faf.12599

[110] Zhang, F., Regular, P. M., Wheeland, L., Rideout, R. M. & Mogan, J. M. Accounting for non-stationary stock–recruitment relationships in the development of MSY-based reference points. *ICES J. Mar. Sci.* **78**, 2233–2243. 10.1093/icesjms/fsaa17 (2021). 10.1093/icesjms/fsaa17

[111] Hamon, K., Ulrich, C. and Kell, L. T. (2007) Evaluation of management strategies for the mixed North sea roundfish fisheries with the FLR framework. MODSIM07—Land, Water and environmental management: Integrated systems for sustainability, in. Proceedings Modelling and Simulation Society of Australia and New Zealand, 2813–2819.

[112] Romagnoni, G. *et al.* Influence of larval transport and temperature on recruitment dynamics of North sea cod (*Gadus morhua*) across spatial scales of observation. *Fish. Oceanogr.* **29**, 324–339. 10.1111/fog.12474 (2020). 10.1111/fog.12474

[113] ICES. Benchmark workshop on Northern Shelf cod stocks (WKBCOD). *ICES Sci. Rep.* **5**(37), 425. 10.17895/ices.pub.22591423 (2023). 10.17895/ices.pub.22591423

[114] ICES. Cod (*Gadus morhua*) in Subarea 4, divisions 6.a and 7.d, and Subdivision 20 (North Sea, West of Scotland, eastern English Channel and Skagerrak). Report of the ICES Advisory Committee, 2023. ICES Advice (2023), cod.27.46a7d20; 10.17895/ices.advice.21840765

[115] Reubens, J. T., Degraer, S. & Vincx, M. The ecology of benthopelagic fishes at offshore wind farms: A synthesis of 4 years of research. *Hydrobiologia* **727**, 121–136. 10.1007/s10750-013-1793-1 (2014). 10.1007/s10750-013-1793-1

[116] Gimpel, A. *et al.* Ecological effects of offshore wind farms on Atlantic cod (*Gadus**morhua*) in the southern North Sea. *Sci. Total Environ.* **878**, 162902. 10.1016/j.scitotenv.2023.162902 (2023). 36934919 10.1016/j.scitotenv.2023.162902

[117] Jacobsen, N. S., Marshall, K. N., Berger, A. M., Grandin, C. & Taylor, I. G. Climate-mediated stock redistribution causes increased risk and challenges for fisheries management. *ICES J. Mar. Sci.* **79**, 1120–1132. 10.1093/icesjms/fsac029 (2022). 10.1093/icesjms/fsac029

[118] Craig, J. K. & Link, J. S. It is past time to use ecosystem models tactically to support ecosystem-based fisheries management: Case studies using Ecopath with Ecosim in an operational management context. *Fish Fish* **24**, 381–406. 10.1111/faf.12733 (2023). 10.1111/faf.12733

