# Supplementary material for: Robust fisheries management strategies under deep uncertainty
Source: Sci Rep. 2024 Jul 23;14:16863. doi: 10.1038/s41598-024-68006-5 (PMC11266645; doi:10.1038/s41598-024-68006-5)
Supplement: Supplementary file 1 — Supplementary Information. [file 41598_2024_68006_MOESM1_ESM.docx]

**Robust fisheries management strategies under deep uncertainty – Supplementary material**

Jan Conradt^1*^, Steffen Funk^1^, Camilla Sguotti^1,2^, Rudi Voss^3,4^, Thorsten Blenckner^5^, Christian Möllmann^1^

^1^Institute of Marine Ecosystem and Fishery Science, Universität Hamburg, Große Elbstraße 133, 22767 Hamburg, Germany

^2^Department of Biology, University of Padova, Via U. Bassi 58/B, 85121 Padova, Italy.

^3^German Centre for Integrative Biodiversity Research (iDiv), Puschstraße 4, 04103 Leipzig, Germany

^4^Center for Ocean and Society (CeOS), Christian-Albrechts-University Kiel, Neufeldtstraße 10, 24118 Kiel, Germany.

^5^Stockholm Resilience Centre, Stockholm University, Frescativägen 8, 10691 Stockholm, Sweden

^*^Institute of Marine Ecosystem and Fishery Science

Universität Hamburg

Grosse Elbstrasse 133

22767 Hamburg, Germany

[jan.conradt@uni-hamburg.de](mailto:jan.conradt@uni-hamburg.de)

+49 40 42838 6658

**Supplementary Methods 1: Description of the population model**

General overview

We used a population model that is essentially a forward simulation of the catch-at-age stock-assessment model [1], which itself is based on the founding theories of fish population dynamics by [2]. The population model is climate-forced via a stock-recruitment model that is driven by sea-surface temperature (SST) in addition to spawning-stock biomass (SSB) A similar setup was originally used by [3] to model the climate-driven population dynamics of sardine and anchovy stocks in the California-Current. In our case, we omitted the addition of random Gaussian noise to the recruitment process ecosystem used by those authors. An overview is given in Suppl. Fig. M1.1.

We started model projections from the year 2029 (with 2030 as the first year of projected stock) and assuming a population equalling MSY B_trigger_ (97.78 kt) in terms of SSB as a result of a hypothetical successful stock recovery until that year. Distribution of stock size (in numbers) over age classes was assumed to equal the median distribution pattern over years 2014-2018.


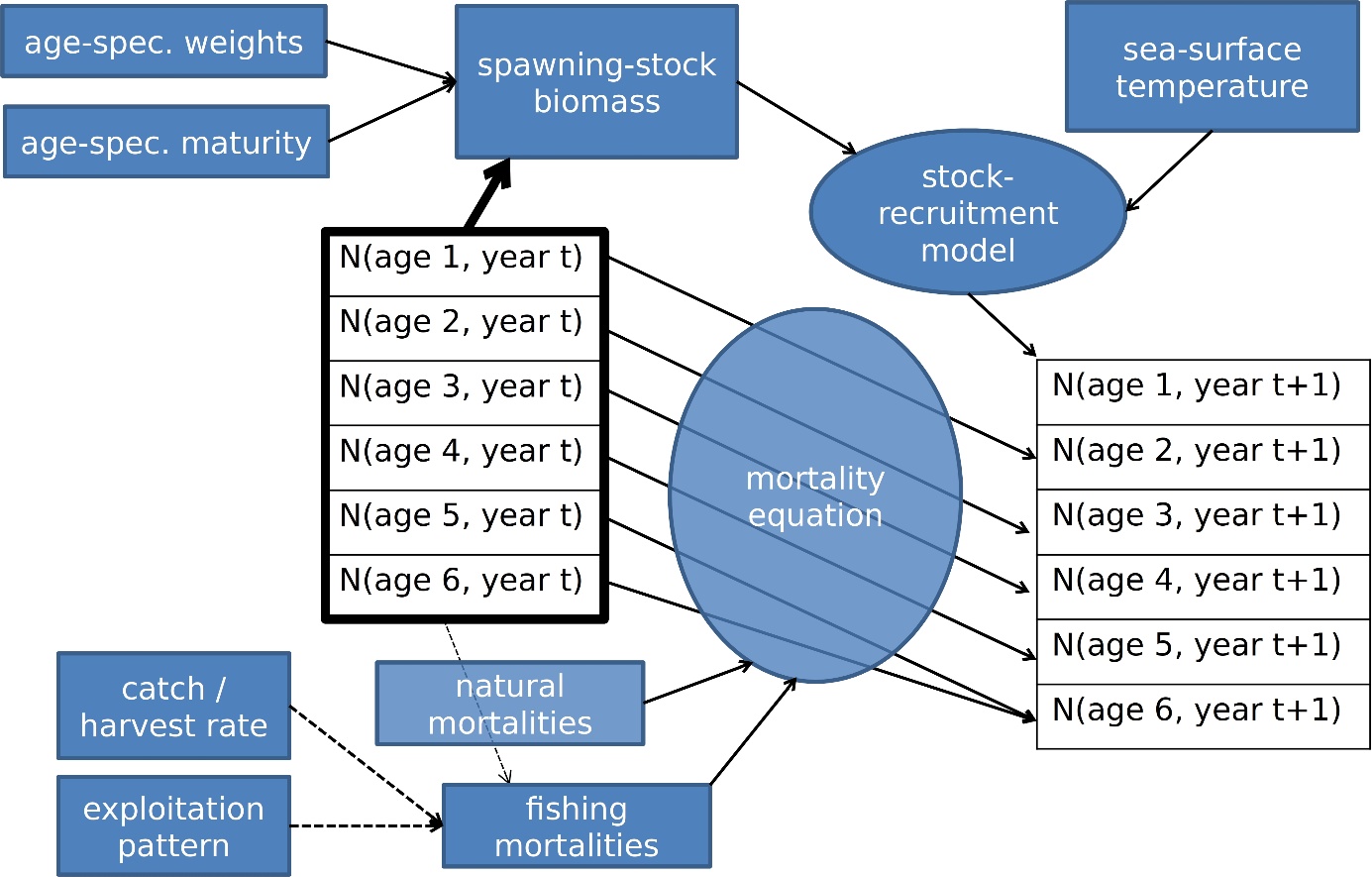


**Supplementary Figure M1.1.** Overview of the population model. Catch (or harvest rate converted to catch via stock biomass) and population numbers are used to estimate fishing mortality, which in conjunction with natural mortality reduces fish in a given cohort in the course of one year. The year-class strength (number of age-1 fish) is estimated via the stock-recruitment function, which integrates SSB (calculated from population numbers and age-specific weights and maturity rates) and SST

Calculation of population numbers

For each age class (five plus the plus-group, which collects fish aged six years and older) and each year, the number of fish surviving to the next year and age-class is calculated via the mortality equation, which integrates natural mortality (death through predation or other natural courses) and fishing mortality (F) (death through fishing) (Suppl. Eq. M1.1). Both the fifth age-class and the plus-group of a given year contribute to the number of fish in the plus group of the following year. The number of age-1 fish added to the population is predicted from SSB and SST via the stock-recruitment function (Supplementary Methods 2).

$N_{t+1,a+1}=N_{t,a}e^{-(F_{t,a}+M_{t,a})}$for $a\in[2, 5]$

$N_{t+1,A}=N_{t,a}e^{-(F_{t,a}+M_{t,a})}+N_{t,A}e^{-(F_{t,A}+M_{t,A})}$for $a=A-1=5$

Supplementary Equation M1.1. Mortality equation. N = population number, F = fishing mortality, M = natural mortality, a = age class, t = time (1 year)

Exploitation pattern

Forcing the population model with catch requires calculating age-specific catch from a total catch in kilo-tonnes. The application of an age-specific exploitation pattern for the distribution of catches over age classes is thus necessary. To determine an exploitation pattern, for each projection year, we initially calculated a reference catch in numbers from age-specific stock sizes after half a year of natural mortality only and age-specific catchability (Suppl. Eq. M1.3), as given by the ratio of F to the maximum F over age classes (Suppl. Eq. M1.2). The latter was calculated as the median over the last five used assessment years (2014-2018).

$$\gamma_{a}=\frac{F_{a}}{\max_{1\to A} \boldsymbol{F}}$$

Supplementary Equation M1.2. Calculation of age-specific catchability. γ = catchability, F = fishing mortality, median over assessment years 2014-2018, a = age class, A = number of age classes

$$C_{{ref}_{a,t}}^{N}=\gamma_{a}N_{a,t}e^{-{0.5M}_{a}}$$

Supplementary Equation M1.3. Calculation of reference catch. $C_{ref}^{N}$= reference catch in numbers, γ = catchability, N = size of the age class at the beginning of the year, M = natural mortality, a = age class, t = time in years

The reference catch was multiplied with weight in the catch (median over the last five used assessment years) to obtain age-specific reference catch in kilo-tonnes (Suppl. Eq. M1.4).

$$C_{{ref}_{a,t}}^{W}=C_{{ref}_{a,t}}^{N}w_{a}^{c}$$

Supplementary Equation M1.4. Calculation of reference catch in weight. $C_{ref}^{W}$= reference catch in weight, $C_{ref}^{N}$= reference catch in numbers, w^c^ = weight in the catch, median over assessment years 2014-2018, a = age class, t = time in years

Age-specific reference catch in weight was then used to calculate an exploitation pattern that allows spreading a given total catch over age classes (Suppl. Eq. M1.5).

$$E_{a,t}=\frac{C_{{ref}_{a,t}}^{W}}{\sum_{a=1}^{A} C_{{ref}_{a,t}}^{W}}$$

Supplementary Equation M1.5. Calculation of exploitation pattern for generating age-specific catch in weight. E = exploitation pattern (relative value), $C_{ref}^{W}$= reference catch in weight, a = age class, t = time in years, A = number of age classes

It was verified that this approach, when calculating F from age-specific catches (see below) recreates the current F pattern in the projections when age class strengths support the intended level of catch. When some age classes were depleted (i.e., contained zero individuals in the model context), the procedures above yielded notably higher relative exploitation for the remaining classes, leading to much-increased realized catchabilities compared to those resulting from Suppl. Eq. M1.2. This change bears some lack of realism from a fisheries logistics point of view, but is required in our model in order to stress-test a given level of catch. Adopting a general scheme of correcting for a more realistic exploitation pattern could lead to a high discrepancy between intended- and realized catch, and thus reduce the meaningfulness of our analyses. The changed exploitation pattern in the model could be interpreted, to some extent, as fishers adapting their strategies (e.g. spending more days at sea) in order to maintain annual catch levels.

Estimation of F

Stock exploitation, was integrated into the population model by estimating age-specific fishing mortalities from catch or harvest rate. Since mortalities are calculated on the level of population numbers, not biomass, catch values given in tonnes were converted to catches in numbers (in thousands) via age-specific weights-in-the-catch (Suppl. Eq. M1.6, top). In our definition, harvest rate is the percentage of stock biomass available after half a year of natural mortality and individual growth; hence we use average individual weight-in-the-catch to calculate that biomass, and apply the harvest rate on this value to derive catch. Age-specific catch was calculated by multiplying total catch by the respective value of the exploitation-pattern (Suppl. Eq. M1.6).

$$C_{a}^{N}=\frac{E_{a}C^{W}}{w_{a}^{c}}$$

$$C_{a,t}^{N}=\frac{E_{a}h\sum_{a=1}^{A} N_{a,t}e^{-{0.5M}_{a}}w_{a}^{c}}{w_{a}^{c}}=\frac{E_{a}C^{W}}{w_{a}^{c}}$$

Supplementary Equation M1.6. Calculation of age-specific catch in numbers. Top, calculation for catch-based projections; bottom, calculation for harvest-rate-based projections. C^N^ = catch in numbers, C^W^ = catch in tonnes, E= exploitation pattern (relative value), h = harvest rate, N = population number, a = age class, w^c^ = weight in the catch, t = time (1 year)

In case age-specific catch was larger than the age-specific number of fish in the stock, reduced by half a year of natural mortality, age-specific catch was set to that number (Suppl. Eq. M1.7). In this case, F is infinite, and the cohort collapses. The procedure results in a lower-than-intended total catch (Suppl. Eq. M1.7); and often in the collapse of the entire stock and catch reduction to zero kt within few years or in the recovery of the catch to intended levels after a few years. A lower-than-intended harvest rate can also occur, when the age-specific catches resulting from total catch (derived from stock biomass and harvest rate) exceed the population size of the individual age classes. In any case, stabilization on a lower-than-intended exploitation level was prevented (Supplementary Results 1). Analyses of the projection outcomes refer to the non-adjusted, “intended”, catch- and harvest-rate levels, as these are the exploitation levels originally intended to be applied over the whole time series, and as realized exploitation varies with the uncertain scenarios simulated. The percentage of uncertain scenarios in which intended exploitation exceeded realized exploitation was calculated (employing methods used in risk analysis; see *Material & Methods* / *Exploratory Modeling*). It increased steadily with intended catch up to a level of 80-90 % at maximum intended catch (with no catch level yielding a risk of zero %), and remained relatively close to zero over the full range of harvest rate employed (Suppl. Fig. R1.2).

$$C_{a,t}^{N}=min(C_{a,t}^{N}, N_{a,t}e^{-0.5M_{a,t}})$$

$$C_{t}^{W}=\sum_{a=1}^{A} C_{a,t}^{N}w_{a}^{c}$$

Supplementary Equation M1.7. Calculation of realized age-specific (top) and total catches (bottom). C^W^= catch in tonnes, C^N^= catch in thousands, N = population number, a = age class, w^c^ = weight in the catch, M = natural mortality, t = time (1 year)

Age-specific fishing mortalities were estimated by minimizing the squared difference between observed catch and the prediction of the catch equation (Suppl. Eq. M1.8), using the “nlminb” optimizer in R. The initial estimate of F required for starting the optimizer was set to the F estimated via the Pope equation [4]. The Pope equation is a simplified procedure for calculating population numbers that assumes that all annual catch is taken in the middle of the year, and that only natural mortality reduces the stock before and after (Suppl. Eq. M1.9). It can thus be used to estimate population numbers without the need to calculate F, but gives slightly biased results. F can be estimated afterwards by re-arranging the mortality equation, and inserting the population number estimated via the Pope equation (Suppl. Eq. M1.10).
In cases where the optimizer did not converge on a final estimate, we supplied the Pope estimate instead.

$$C_{a,t}^{N}=N_{a,t}(1-e^{-\left( F_{a,t}+M_{a,t} \right)})\frac{F_{a,t}}{F_{a,t}+M_{a,t}}$$

Supplementary Equation M1.8. Catch equation. C^N^ = catch in numbers, N = population number, F = fishing mortality, M = natural mortality, a = age class, t = time (1 year)

$$N_{a+1,t+1}\approx{((N}_{a,t}e^{-{0.5M}_{a,t}})-C_{a,t}^{N})e^{-{0.5M}_{a,t}}$$

Supplementary Equation M1.9. Pope’s (1972) approximation of the mortality equation. C_N_ = catch in numbers, N = population number, F = fishing mortality, M = natural mortality, a = age class, t = time (1 year)

$$F_{a,t}=-(\left( logN_{a+1,t+1}-logN_{a,t} \right)+M_{a,t})$$

Supplementary Equation M1.10. Mortality equation solved for fishing mortality. N = population number, F = fishing mortality, M = natural mortality, a = age class, t = time (1 year)

Model constants

Parameters of the population model aside from those of the stock-recruitment model were set to the median of the values of the years 2014 to 2018, as given by the ICES stock assessment [5]. These are the age-specific natural mortalities, age-specific weights in the stock, age-specific weights in the catch age-specific maturity rates (relative amount of spawning fish) and age-specific catchabilities (Suppl. Tab. M1.1). Age-specific weights in the catch were given for all age-classes caught; weights for age classes 6 and higher were summarized as plus-group weight by taking the median weight over these age-classes. The final two assessment years (2019 and 2020) were omitted from calculating average biological parameter values due to generally higher uncertainties in later assessment years [6]. All parameters mentioned above were kept constant in value among years in the projections.

The population model also requires an age-resolved start population for the initial year as input. Since we assumed recovery of the stock until 2029, this was set to population numbers calculated from MSY B_trigger_, the above-mentioned estimates of weight in the stock and maturity and the relative distribution of weights over age classes: We assumed that the current relative proportions of age classes to the total population size remain unchanged until 2030.

In a first step, age-specific initial SSB was calculated by multiplying MSY B_trigger_ with the ratio of current age-specific SSB values to total current SSB (Suppl. Eq. M1.12).

$${SSB}_{a}^{ini}={MSYB}_{trigger}\frac{w_{a}^{s}m_{a}N_{a}}{\sum_{a=1}^{A} w_{a}^{s}m_{a}N_{a}}$$

Supplementary Equation M1.12. Calculation of age-specific initial SSB. SSB^ini^ = initial SSB, w^s^ = weight in the stock (median over assessment years 2014-2018), m = maturity (median over assessment years 2014-2018), N = age-specific population size (median over assessment years 2014-2018), a = age class, A = number of age classes

Age-specific SSB was then used to calculate age-specific initial population numbers (2030 start-of-the-year), by dividing the former by the age-specific product of weight and maturity (Suppl. Eq. M1.13).

$$N_{a}^{ini}=\frac{{SSB}_{a}^{ini}}{w_{a}^{s}m_{a}}$$

Supplementary Equation M1.13. Calculation of initial age-specific population size. N^ini^ = initial population size, SSB^ini^ = initial SSB, w_s_ = weight in the stock ((median over assessment years 2014-2018), m = maturity (median over assessment years 2014-2018), a = age class

The first simulated year of catch was 2029, and the stock projection began in the year 2030.

Supplementary Table M1.1. Population-model parameters

| parameter | age 1 | age 2 | age 3 | age 4 | age 5 | age 6+ |
| --- | --- | --- | --- | --- | --- | --- |
|  |  |  |  |  |  |  |
| natural mortality | 1.192 | 0.943 | 0.476 | 0.362 | 0.363 | 0.363 |
| weight in the stock [kg] | 0.064 | 0.551 | 1.756 | 3.276 | 5.143 | 7.215 |
| weight in the catch [kg] | 0.366 | 0.927 | 2.138 | 3.803 | 5.624 | 9.218 |
| maturity rate | 0.017 | 0.145 | 0.441 | 0.761 | 0.865 | 1.000 |
| catchability | 0.105 | 0.573 | 0.968 | 0.927 | 1.000 | 0.671 |
| initial population number [10^3^] | 188928.45 | 95577.65 | 24690.36 | 9383.31 | 5500.43 | 3181.22 |

**Supplementary Methods 2: Extended description of stock-recruitment models**

Theoretical foundations

The Beverton-and-Holt model [7,8] (Suppl. Eq. M2.1) contains two parameters related to stock biomass: the parameter *α* describes a positive effect of the spawning-stock biomass (SSB) on recruitment strength, while the parameter *β* summarizes effects that relate to the carrying capacity of the ecosystem; these include i.a. competition for food. Effectively, this means that recruitment strength increases asymptotically with increasing SSB. An additional parameter, *γ*, describes environmental effects that act negatively on recruitment; these are usually related to physical effects affected by climate change, e.g. temperature.

$$R_{t+1}=N_{t+1,1}=e^{-\gamma E_{t}}\frac{\alpha{SSB}_{t}}{1+\beta{SSB}_{t}}$$

Supplementary Equation M2.1. Environmental Beverton-and-Holt (Hilborn & Walters, 1992) stock-recruitment-model equation. R = recruitment, N = population number, SSB = spawning-stock biomass, E = environmental variable. Same as Eq. 1 (top)

The Ricker model [9,10] (Suppl. Eq. M2.2) contains a similar set of parameters, with *α* describing a positive and *β* describing a negative effect of stock size on recruitment strength. However, unlike the Beverton-and-Holt model, it assumes that recruitment strength is negatively affected by high SSB, such that highest recruitment strength is achieved at intermediate levels of SSB.

$$R_{t+1}=N_{t+1,1}={\alpha SSB}_{t}e^{-\beta{SSB}_{t}-\gamma E_{t}}$$

Supplementary Equation M2.2. Environmental Ricker (1975) stock-recruitment-model equation. R = recruitment, N = population number, SSB = spawning-stock biomass, E = environmental variable. Same as eq. 1 (bottom)

Calculation of spawning-stock biomass

Annual SSB was calculated by summing, over all age classes, the product of number of fish in a given age class and age-specific weight and maturity rate (Suppl. Eq. M2.3). This was then used as input for the SR model to calculate the number of age-1 fish in the following year.

$${SSB}_{t}=\sum_{a}^{A} N_{a,t}w_{a,t}m_{a,t}$$

Supplementary Equation M2.3. Calculation of annual spawning-stock biomass (SSB). N = population number, w = weight, m = maturity rate, a = age class, A = number of age classes, t = year

Fitting of the stock-recruitment models

Both the Ricker and the Beverton-and-Holt model were fitted with the nlsLM optimizer that applies the Levenberg-Marquardt algorithm to non-linear least-squares regression [11]. The original Beverton-Holt- and Ricker equations (Suppl. Eqs. M2.1-2) were re-arranged (Suppl. Eq. M2.4) to allow i) for a formulation closer to that of a linear equation, which can alleviate the fitting procedure, and ii) for fitting the logarithms of the SSB-related parameters (*α* and *β*). These parameters have no biological meaning when being negative. Determining a mean estimate and standard deviation of the logarithms of these parameters thus ensures that no negative values will result when sampling from the uncertainty range.

The re-arranged equations necessitate a back-transformation (exponentiation and additional multiplication with SSB in the Ricker model) of the model predictions to obtain recruitment values.

$$\log\left( R_{t+1} \right)=-\gamma{SST}_{t}+lg\alpha+\log\left( {SSB}_{t} \right)-\log\left( 1+e^{lg\beta+log({SSB}_{t})} \right)$$

$$\log\left( \frac{R_{t+1}}{{SSB}_{t}} \right)=\alpha^{'}-e^{\beta^{'}}{SSB}_{t}-\gamma{SST}_{t}$$

where $lg\alpha=log(\alpha)$ and $lg\beta=log(\beta)$

Supplementary Equation M2.4. Beverton-Holt- (top) and Ricker (bottom) SR functions, re-arranged equations. For details see Suppl. Eq. M2.1-2. Note that lgα and lgβ are treated as the optimizable parameters, instead of α and β directly

Final parameter estimates for both stock-recruitment models are given in Suppl. Tab. M2.1.

Supplementary Table M2.1. Final parameter estimates and standard errors for the two stock-recruitment models

| model | parameter | estimate | standard error |
| --- | --- | --- | --- |
|  |  |  |  |
| Ricker | log(α) | 10.34 | 1.674 |
| Ricker | log(β) | -12.27 | 0.3693 |
| Ricker | γ | 0.7952 | 0.1543 |
| Beverton-Holt | log(α) | 11.29 | 1.939 |
| Beverton-Holt | log(β) | -11.16 | 0.7954 |
| Beverton-Holt | γ | 0.8502 | 0.1616 |

**Supplementary Figure 1: Stock-recruitment relationships – partial-effects of SSB and SST**


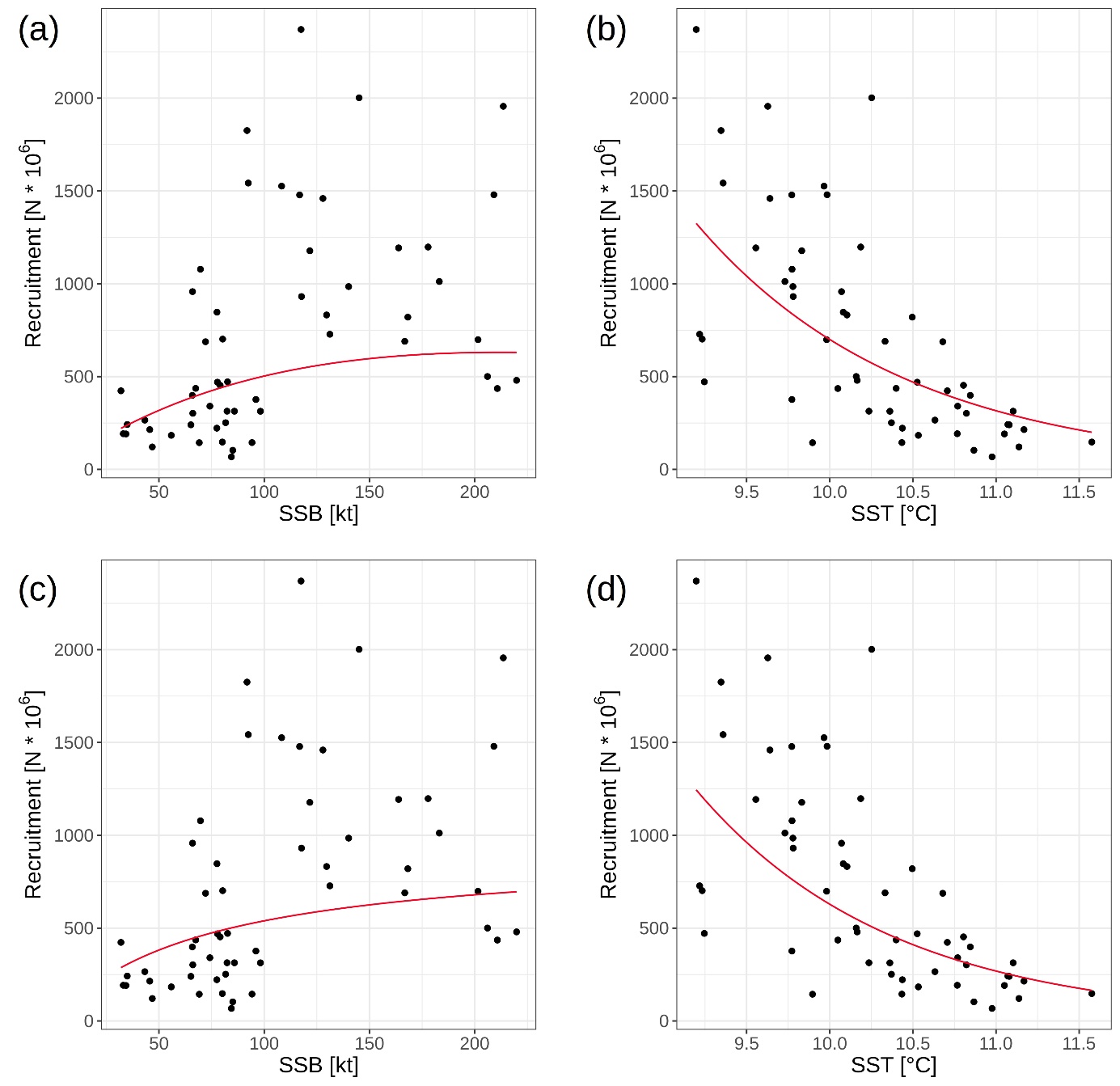


**Supplementary Figure 1.** Partial-dependence plots showing effect strength and direction of SSB and SST on recruitment as modeled with the Ricker (**a**, **b**) and Beverton-Holt (**c**, **d**) stock-recruitment models fitted on the full time series

**Supplementary Methods 3: Trajectories of future SST development**

Trajectories of future SST development were obtained from the output of a regional physical ocean model [12], which were bias-corrected against historical reconstructions of North Sea SST (which had been used in the fitting of the SR models) (NOAA Extended Reconstructed Sea Surface Temperature; ERSST [13]). Bias correction was conducted by calculating the median difference between model hind-cast and early projections, and the ERSST data, and adding the offset to the future projection data [14]. Bias-corrected SST projections are visible in Suppl. Fig. M3.1.

With the initial projection year set to 2029 (with 2029 SSB assumed to equal MSY B_trigger_ and the initial recruitment projection being for 2030), the SST time series were also truncated at the start such as to commence with the 2029 values.


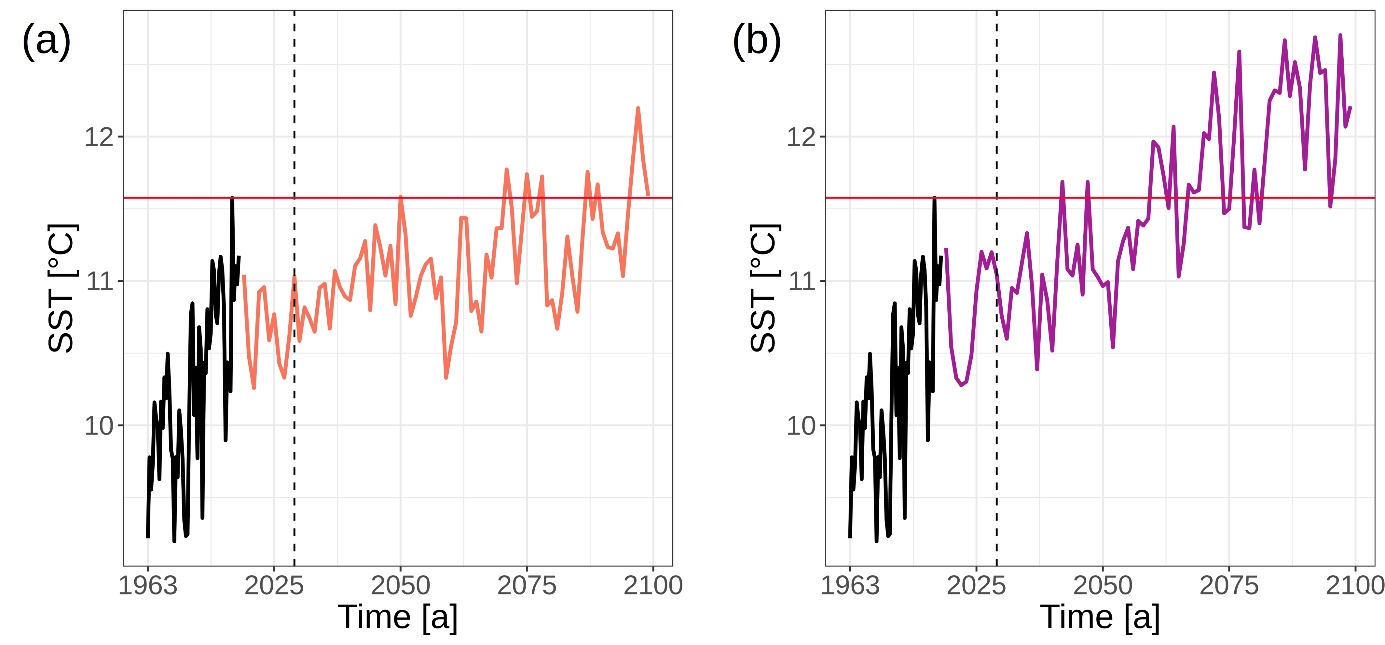


**Supplementary Figure M3.1.** Trajectories of future SST for the RCP4.5 scenario (**a**) and the RCP8.5 scenario (**b**). Red horizontal line denotes maximum SST observed in the past. Dashed vertical line indicates start of the part of the time series used for stock projections

**Supplementary Figure 2: SR relationships resulting from random parameter sampling**


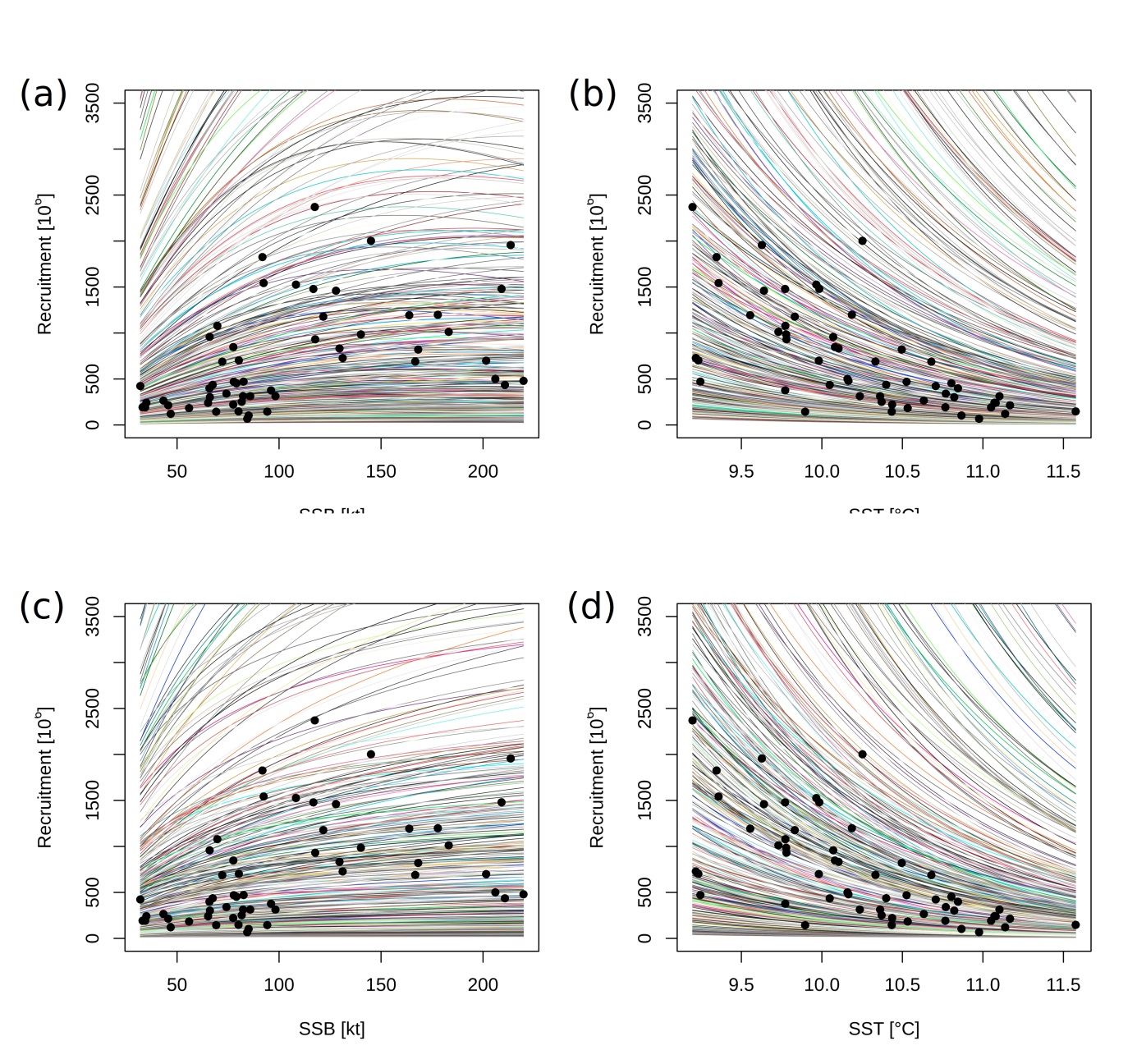


**Supplementary Figure 2.** Assessment-estimated SSB- and recruitment data (dots) and SR relationships resulting from sampling the standard-error range of fitted SR-model parameters. Shown are the partial effects of SSB and SST for each of the 50 random parameterizations, for the Ricker (**a**, **b**) and the Beverton-Holt (**c**, **d**) models

**Supplementary Methods 4: Description of the economic model**

For the calculation of profits, we followed the procedures proposed by [15]): Profits are defined as the difference of revenue and costs (Suppl. Eq. M4.1).

$$P_{t}=r_{t}-\Gamma_{t}$$

Supplementary Equation M4.1. Calculation of profits. P = profits, r = revenue, Γ = costs, t = time

Catch revenue is calculated by assigning caught fish to a specific weight-class associated with a distinct price per weight (as obtained from the German federal office for agriculture and food [16]). In our study, weights were set to the average age-specific weights in the catch for the period 2014-2018, thus each age class was assigned to one price level for the entire projected period (Suppl. Tab. 1). Revenue is then the catch in money summed over all age classes (Suppl. Eq. M4.2). We used the realized catch, i.e. the catch corrected not to exceed population numbers (see Supplementary Methods 1) in revenue calculation.

$$r_{t}=\sum_{a=1}^{A} C_{a,t}^{w}*p_{a}*1000 \mathrm{where} p_{a}=p_{\omega} \mathrm{for} {w_{c}}_{a}\in[\min W_{\omega},\max W_{\omega})$$

Supplementary Equation M4.2. Calculation of revenue. r = revenue, t = time, a = age class, C^w^ = catch in weight, p = price (€ per kg), w_c_ = weight in the catch, ω = weight class. Multiplication with 1000 converts catch in tonnes to catch in kg

The activity of catching also incurs costs, which are positively related to the amount of realized catch (due to e.g. wear of material and labor that increase with the amount of fish caught) (Suppl. Eq. M4.3).

$$\Gamma_{t}=qC_{t}^{w}*1000$$

Supplementary Equation M4.3. Calculation of costs. Γ = costs, q = baseline cost factor, equals 1.21 € per kg, C^w^ = catch, t = time in years. Multiplication with 1000 converts catch in tonnes to catch in kg

To calculate the profitability reference point, profits were similarly calculated for the period of observed data, using catchability coefficients calculated specifically for each year and price-class assignments based on the actual average reported weights.

**Supplementary Table 1: Input parameters for economic model**

Supplementary Table 1. Price for weighted fish

| age class | weight-at-age in the catch [kg] | weight class [kg] | price [Euros * kg^-1^] |
| --- | --- | --- | --- |
|  |  |  |  |
| 1 | 0.37 | 0.3 to 1.0 | 1.500 |
| 2 | 0.93 | 0.3 to 1.0 | 1.500 |
| 3 | 2.14 | 2.0 to 4.0 | 2.653 |
| 4 | 3.80 | 2.0 to 4.0 | 2.653 |
| 5 | 5.62 | 4.0 to 7.0 | 3.133 |
| 6+ | 9.22 | > 7.0 | 1.606 |

**Supplementary Figure 3: Past profits generated by the North Sea cod stock**


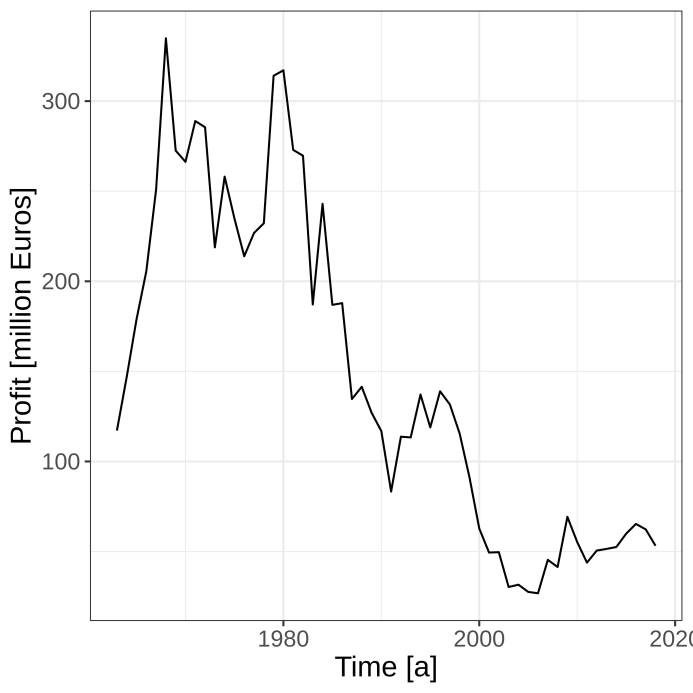


**Supplementary Figure 3.** Past profits generated by North Sea cod for the period 1963-2018. Profits calculated using modeling approach presented by Schenk *et al.* (2023) (see Supplementary Material 7)

**Supplementary Figure 4: Median trends of recruitment and SSB for different climate scenarios**


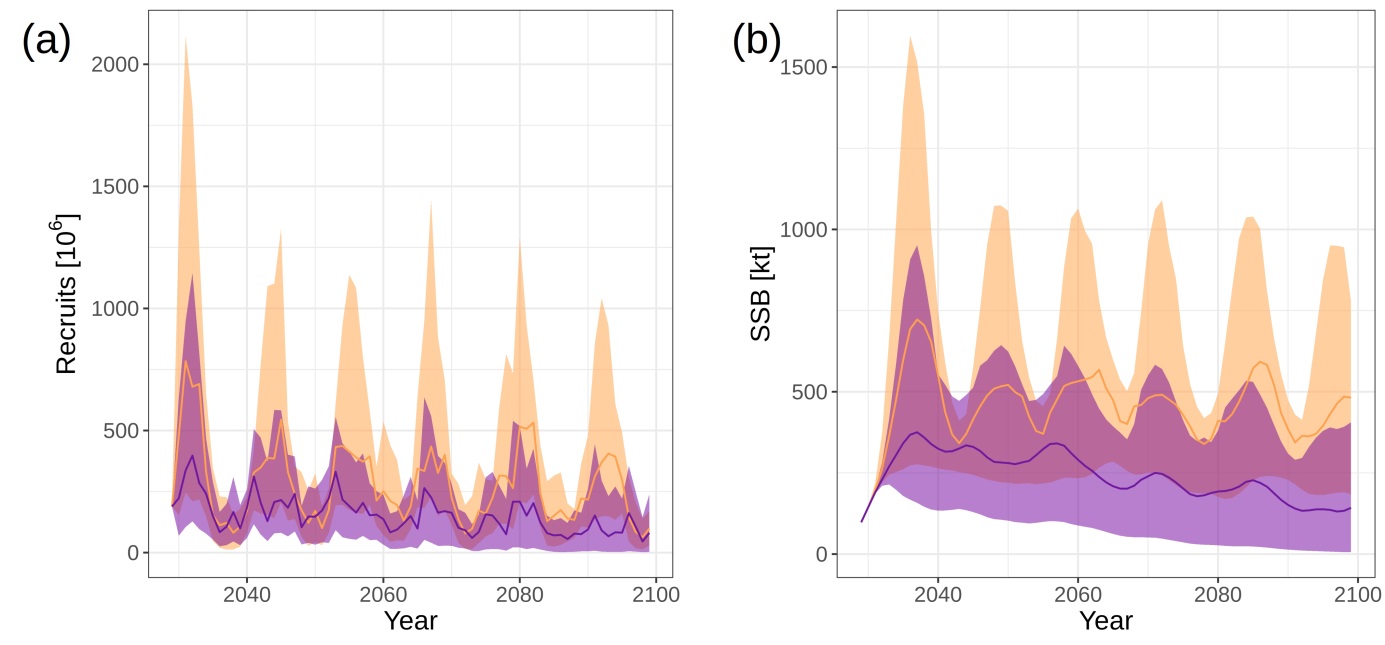


**Supplementary Figure 4.** Projected recruitment (**a**) and SSB (**b**) under RCP4.5 (yellow) and RCP8.5 (blue). Line shows median. Shading represents range between 25- and 75-% percentiles

**Supplementary Methods 5: Determining catch- and harvest-rate limits for sampling ranges**

Lower limits for the ranges of catch and harvest rate that decision alternatives were sampled from were set to zero kt or %, respectively.

The upper limit of catch was set to current stock biomass, which was approximated as sum over population numbers derived from MSY B_trigger_ (see Supplementary Methods 1), reduced by half a year of natural mortality (medians over 2014-2018) and multiplied with weights in the catch (medians over 2014-2018) (Suppl. Eq. M5.1). This value represents a natural limit, as catch cannot exceed initial stock size in the first projection year.

$$C_{max}=\sum_{a=1}^{A} w_{a}^{c}N_{a,0}e^{-0.5M_{a}}$$

Supplementary Equation M5.1. Calculation of the upper sampling limit for catch levels. C = catch, N_0_ = initial population size, M = natural mortality, w^c^ = weight in the catch, a = age class, A = number of age classes

The upper limit of harvest rate was initially set to 100 %, equaling the upper limit of the fixed-catch runs in the first projection year. An initial analysis of projection runs conducted while sampling from the range of zero to 100 % harvest rate revealed that a large part of the range yielded clearly unsustainable outcomes over virtually all uncertain scenarios (Suppl. Fig. M5.1) (for details on the analysis procedure see *Material & Methods / Exploratory modeling*). As we were mainly interested in exploring the changing degree of sustainability in response to exploitation and uncertainty, we accordingly set a final upper limit of 25 % harvest rate (a new set of 100 random levels of harvest rate were drawn from this reduced range and used for the final analyses).


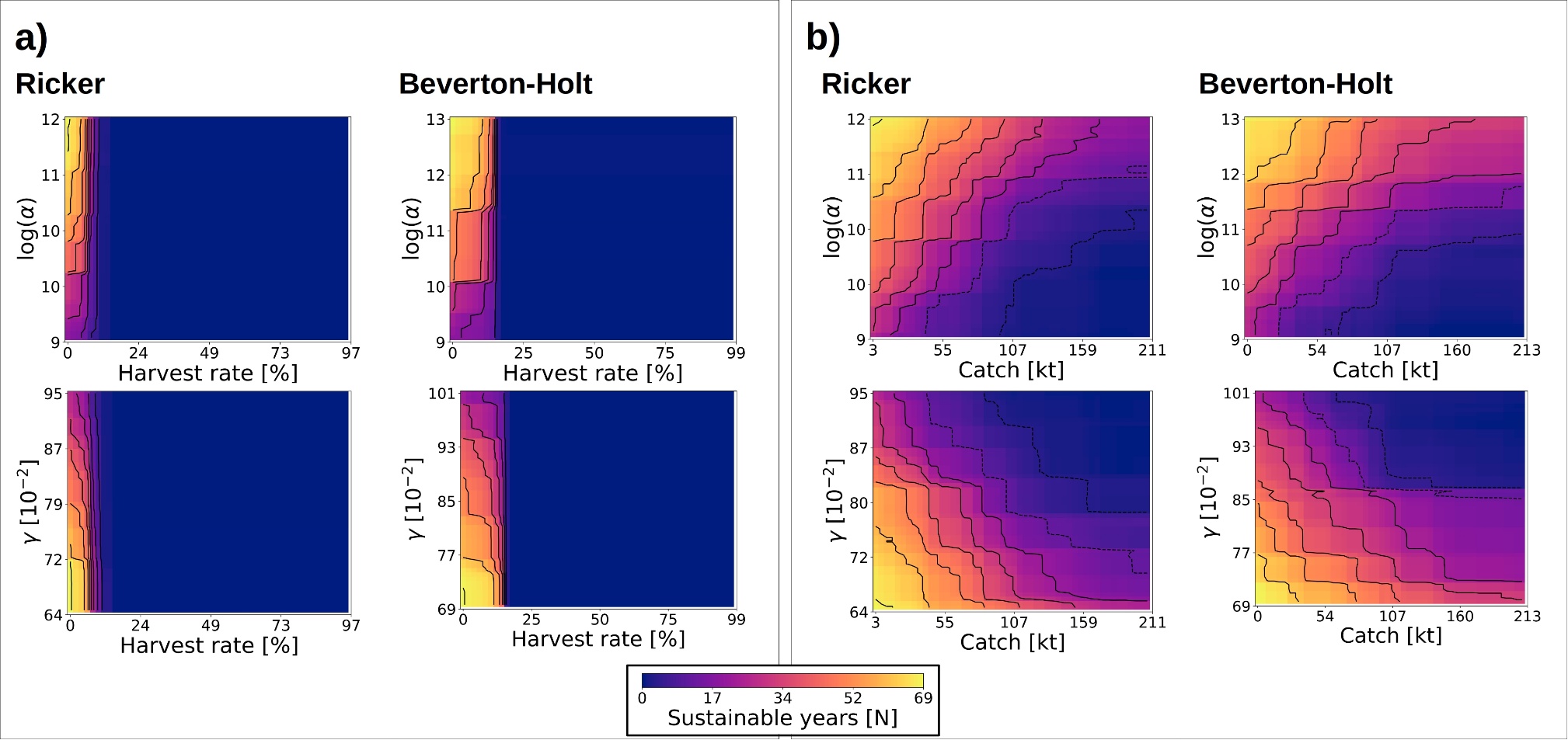


**Supplementary Figure M5.1.** Outcomes of feature scoring for harvest-rate-based projections covering the full range of zero to 100 % harvest rate (**a**) and fixed-catch-based projections (**b**). For further details see Fig. 2 in the main text.

**Supplementary Results 1: Relationship between intended and realized catches**

Realized catch in a model projection conducted with the fixed-catch management scheme can deviate from intended catch (the level of catch taken in the first projection year, which is intended to be taken in all subsequent years, as well). This event occurs when stock size is smaller than the intended level of catch as a result of initial overexploitation and / or insufficient stock productivity. Its occurrence is therefore dependent on uncertainty about the SR relationship.

We calculated the relative amount of instances (uncertain scenarios and years) in which realized catch deviated from intended catch for each bin of catch levels (see *Material & Methods / Exploratory modeling*) and visualized the relationship to obtain information on the amount of occurrences where the results shown relate not to the actual intended catch level but to a lower catch level resulting from forced reduction.

We found that the relative amount of instances in which realized catch deviated from intended catch increased relatively linearly with intended catch, reaching a level of c. 90 % at the highest catch level (Suppl. Fig. R1.1 b). There was no clearly defined range of intended catch where there were no instances of deviation. However, realized catch in general quickly reached zero when it did deviate from intended catch (Suppl. Fig. R1.2 b), indicating that in our model, deviations are caused by relatively quick stock collapse and are terminal, i.e. realized catch does not stabilize on some lower-than-intended level.

Realized harvest rate also differed from intended harvest rate in some instances, though only in the very beginning of the time series, before it returned to the intended level (Suppl. Fig. R1.2 a)


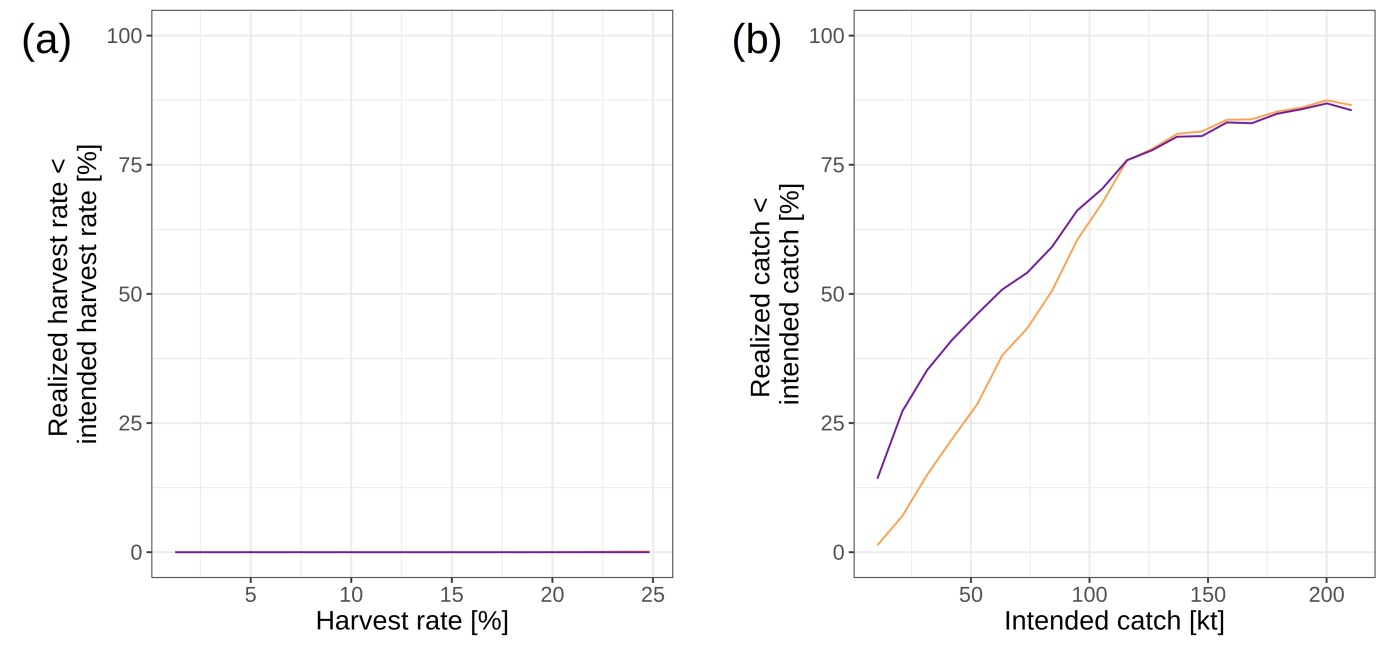


**Supplementary Figure R1.1.** Relative amounts of instances where intended harvest rate exceeded realized harvest rate (**a**) and where intended catch exceeded realized catch (**b**). Yellow line: mid-century projection period (2030-2049); blue line: late-century projection period (2050-2099).


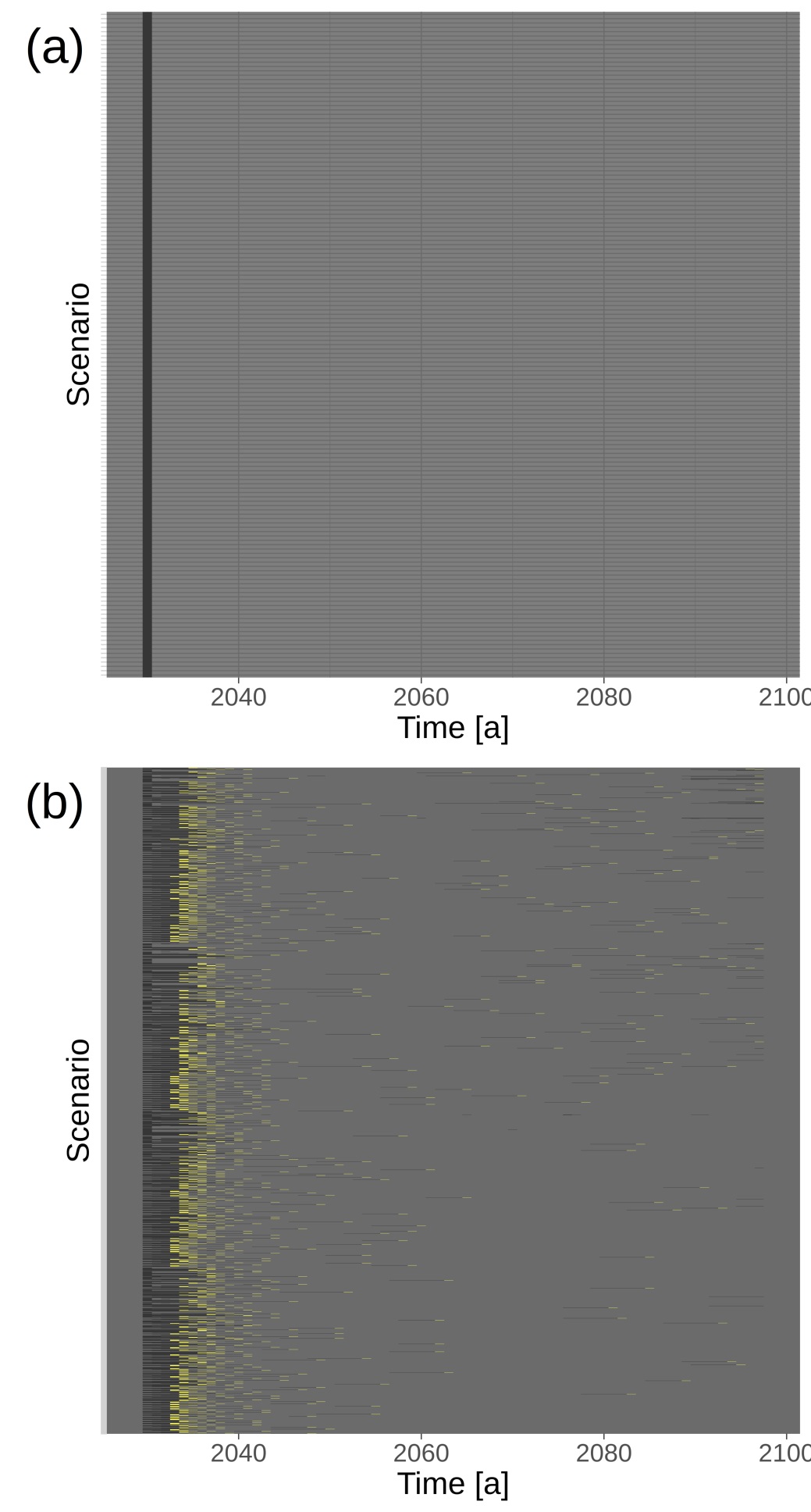


**Supplementary Figure R1.2.** Trajectories of realized catch (**a**) and realized harvest rate (**b**) that resulted in a deviation of realized catch from intended catch or a deviation of realized harvest rate from intended harvest rate, respectively. Black shading indicates that realized exploitation is lower than intended exploitation but is not zero. In (**a**), yellow shading highlights initial year in which catch is zero. In (**b**), there are no occurences of realized harvest rate being zero, as realized harvest rate recovers to the intended level in every case

**Supplementary Methods 6: Determining exploitation at best risk trade-off**

Levels of catch and harvest rate corresponding to the best trade-offs were determined based on two criteria: i) additive risk and ii) absolute difference between 1 and the ratio of profitability- to sustainability risk. The exploitation levels corresponding to the lowest sum of the two criteria were selected as those levels corresponding to best trade-off between sustainability- and profitability risk.

**Supplementary Results 2: Risk dynamics for zero-harvesting scenarios**


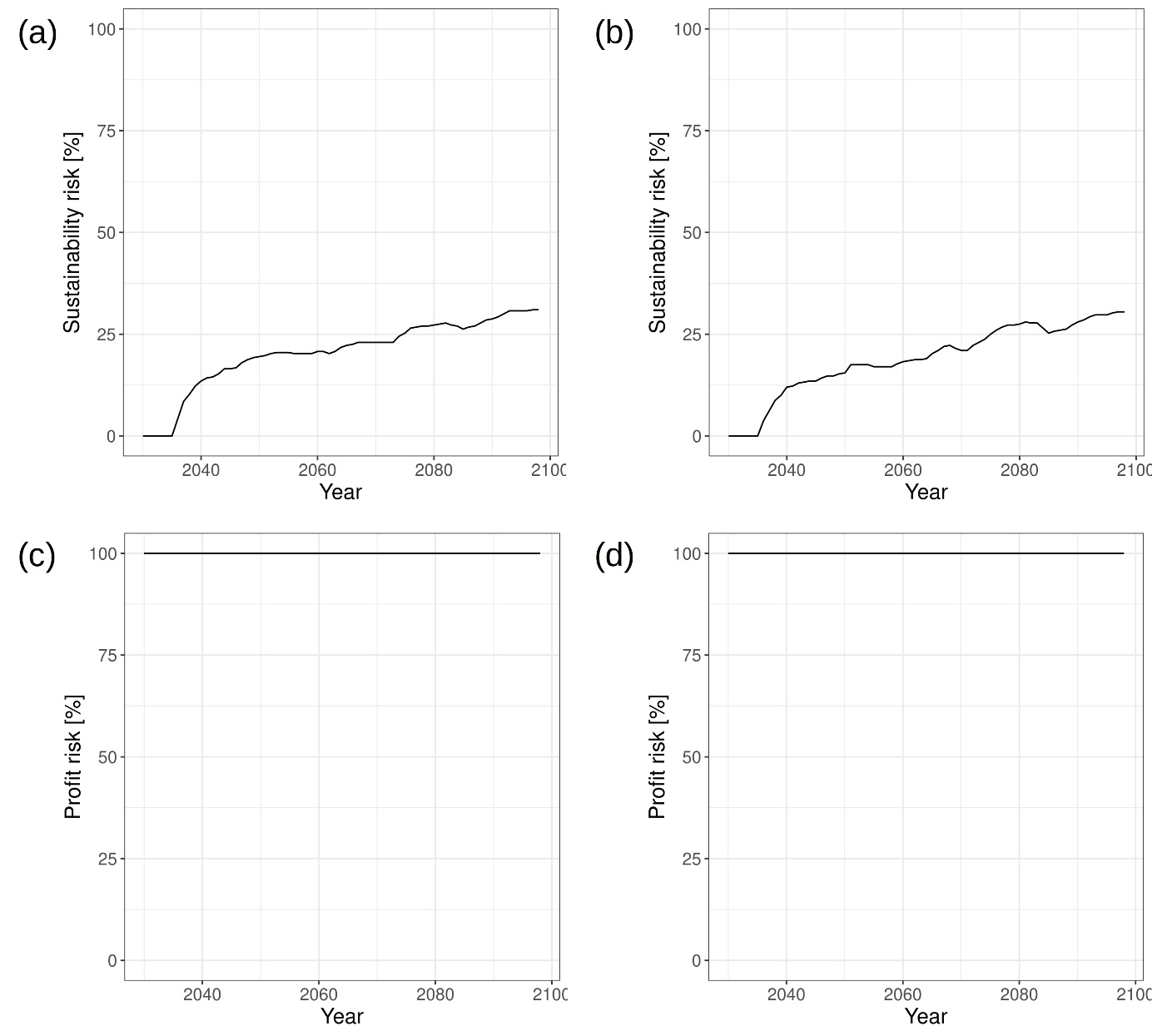


**Supplementary Figure R2.1.** Development of sustainability risk (**a**, **b**) and profitability risk (**c**, **d**) over time for zero-catch scenarios (**a**, **c**) and zero-harvest-rate scenarios (**b**, **d**)

**Supplementary Methods 7: Comparison of environmentally-sensitive SR functions and other SR function candidates**

In addition to the environmentally-sensitive Ricker- [10] and Beverton-Holt [8] functions, we also fitted their environmentally-insensitive analogues [9; 7] and a hockey-stick SR function (with constant recruitment above B_lim_), which is the SR function currently used by the ICES assessment to describe the SR relationship [17] (Suppl. Eq. M7.1).

All SR models were fitted using the “nlsLM()” function of the “minpack.lm” R package [11]. We fitted the hockey-stick model both on the full time series of available SSB and recruitment estimates, and on a limited time series starting at 1998 (as done by ICES [17]), recognized by ICES [17] as a separate recruitment regime. We compared performance of all models via AIC, deviance explained and visual inspection of fit, both for the full time series and the limited time series.

$$R_{t+1}=N_{t+1,1}=e^{-\gamma E_{t}}\frac{\alpha{SSB}_{t}}{1+\beta{SSB}_{t}}$$

$$R_{t+1}=N_{t+1,1}={\alpha SSB}_{t}e^{-\beta{SSB}_{t}-\gamma E_{t}}$$

$$R_{t+1}=N_{t+1,1}=\frac{\alpha{SSB}_{t}}{1+\beta{SSB}_{t}}$$

$$R_{t+1}=N_{t+1,1}={\alpha SSB}_{t}e^{-\beta{SSB}_{t}}$$

$$R_{t+1}=N_{t+1,1}=\left\{ \begin{aligned} \alpha{SSB}_{t} | {SSB}_{t}\leq B_{thres} \\ {\alpha B}_{thres} | {SSB}_{t}>B_{thres} \end{aligned} \right.$$

**Supplementary Equation M7.1**. Environmentally-sensitive and –insensitive SR functions. From top to bottom: Environmentally-sensitive Beverton-Holt function [8], environmentally-sensitive Ricker function [10], classic Beverton-Holt function [7], classic Ricker function [9], hockey-stick function [17]. B_thres_ was set to B_lim_ when fitting for the entire SR time series, and to minimum SSB when fitting for the limited time series

We found that the environmentally-sensitive Ricker- and Beverton-Holt functions yielded markedly higher deviance explained and lower AIC than their climate-insensitive analogues and than the hockey-stick function (Suppl. Tab. M7.1) when fitted on the full time series of SSB and recruitment estimates. The environmentally-sensitive SR functions also showed a better fit in visual inspection (Suppl. Fig. M7.1, Suppl. Fig. M7.2). When evaluating the SR functions for the limited time series of SR data, differences in model performance were less clear, though the environmentally-sensitive SR functions still showed a comparatively good fit (Suppl. Tab. M7.1, Suppl. Fig. M7.3, Suppl. Fig. M7.4). The strong overall performance of the environmentally-sensitive SR models on the full time series, and the lack of marked residual patterns for the predictions on the limited time series (Suppl. Fig. M7.4), indicate that they are adequate for predicting North Sea cod recruitment.

**Supplementary Table M7.1**. Deviance explained of the fitted SR functions

| Function | AIC | Deviance explained |
| --- | --- | --- |
| Environmentally-sensitive Beverton-Holt | 102.39 | 0.47 |
| Environmentally-sensitive Ricker | 103.24 | 0.48 |
| Classic Beverton-Holt | 123.96 | 0.16 |
| Classic Ricker | 123.95 | 0.16 |
| Hockey-stick | 233.45 | 0.12 |
| Hockey stick (fitted and evaluated on limited time series) | - | -0.05 |
| Environmentally-sensitive Beverton-Holt (evaluated on limited time series) | - | -1.96 |
| Environmentally-sensitive Ricker (evaluated on limited time series) | - | -2.02 |
| Classic Beverton-Holt (evaluated on limited time series) | - | -2.93 |
| Classic Ricker (evaluated on limited time series) | - | -2.94 |


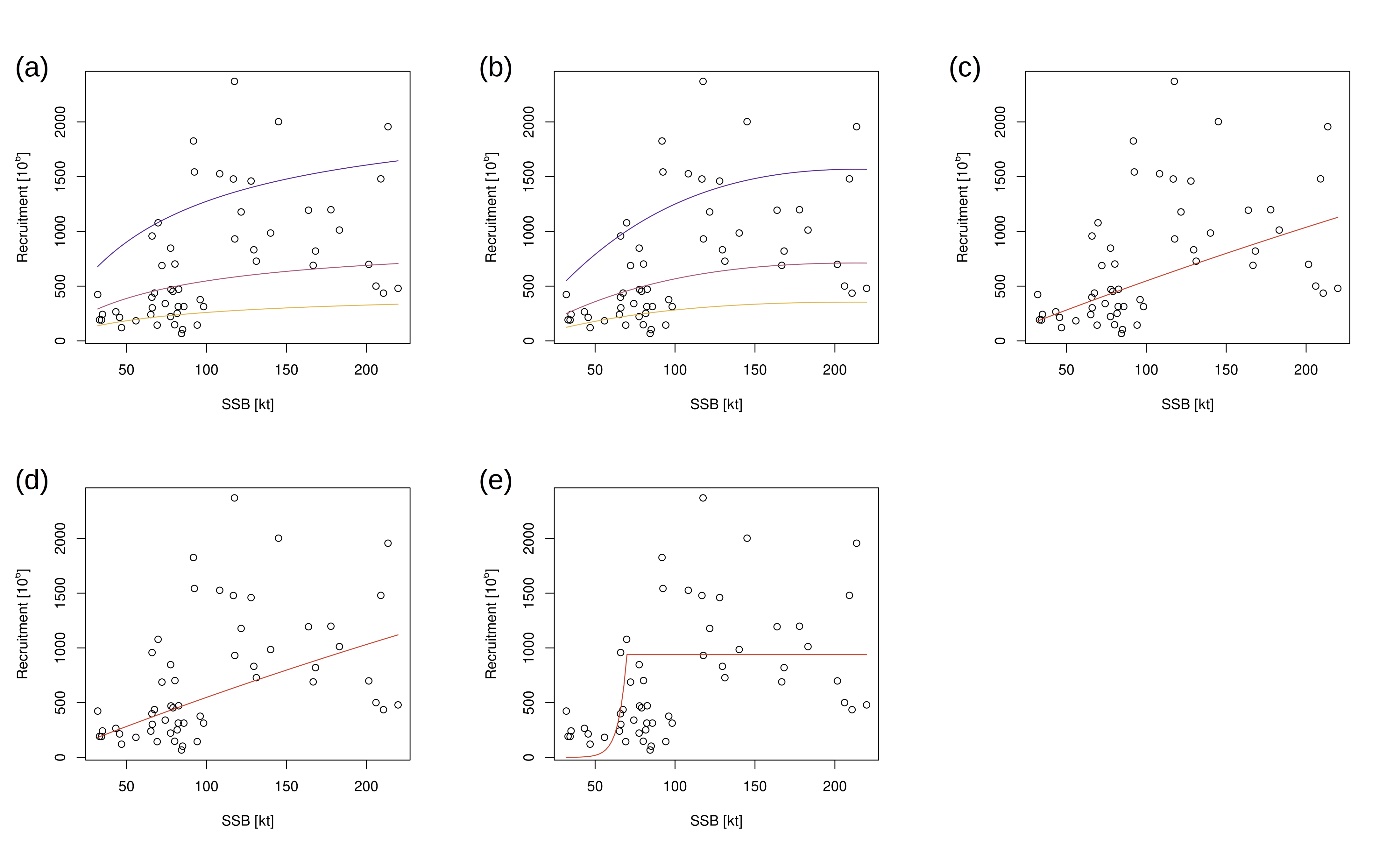


**Supplementary Figure M7.1.** (Partial) effect of SSB on recruitment for environmentally-sensitive and –insensitive SR functions fitted on North Sea cod recruitment and SSB and North Sea SST. (**a**) Environmentally-sensitive Beverton-Holt, (**b**) environmentally-sensitive Ricker, (**c**) classic Beverton-Holt, (**d**) classic Ricker, (**e**) hockey-stick function. Increasingly darker colors in (a) and (b) correspond to predictions with the 5^th^, 50^th^ and 95^th^ percentile of historic North Sea SST


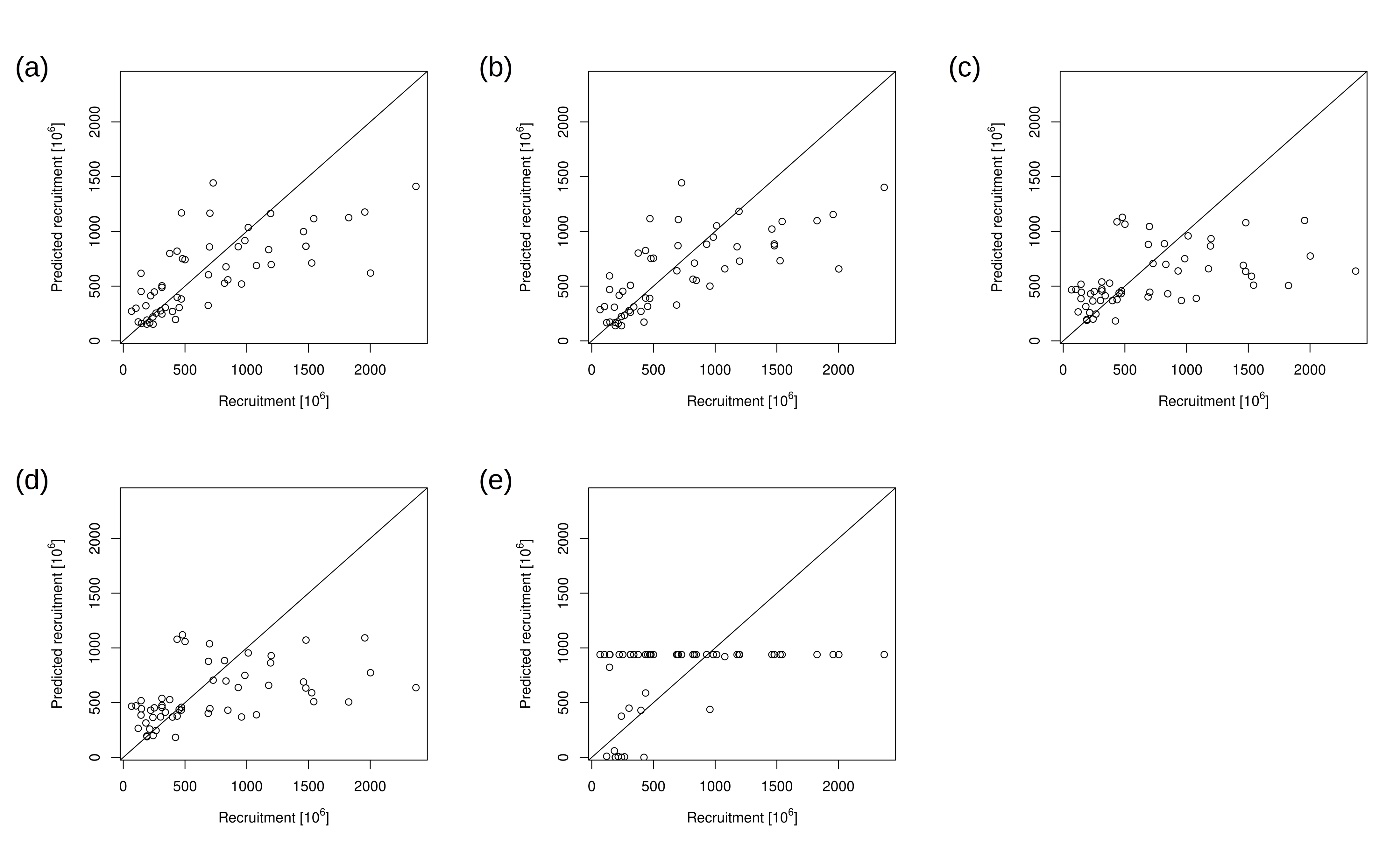


**Supplementary Figure M7.2.** Relationship between predicted recruitment and official recruitment estimates for environmentally-sensitive and –insensitive SR functions fitted on North Sea cod recruitment and SSB and North Sea SST. (**a**) Environmentally-sensitive Beverton-Holt, (**b**) environmentally-sensitive Ricker, (**c**) classic Beverton-Holt, (**d**) classic Ricker, (**e**) hockey-stick function


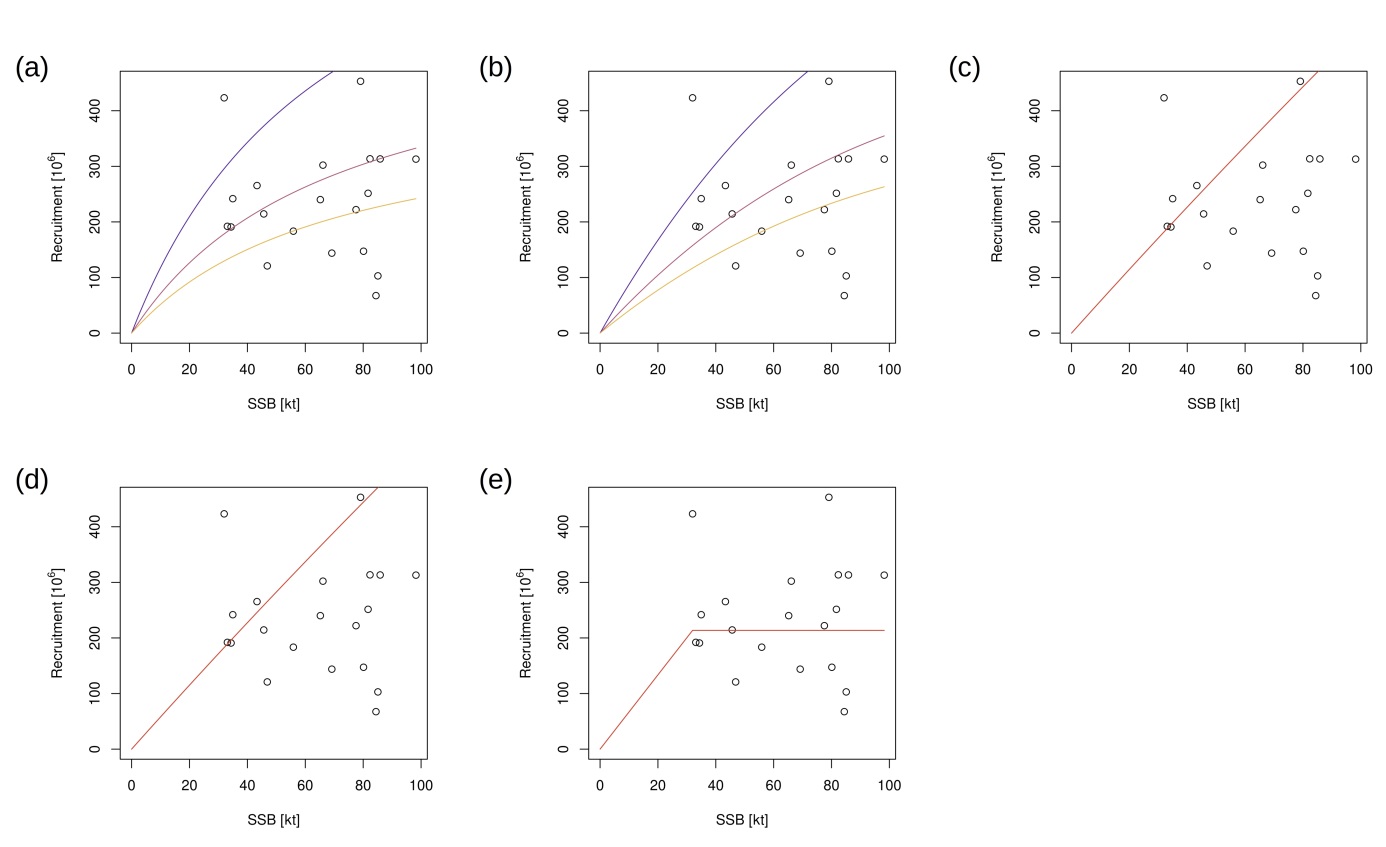


**Supplementary Figure M7.3.** (Partial) effect of SSB on recruitment for environmentally-sensitive and –insensitive SR functions fitted on North Sea cod recruitment and SSB and North Sea SST, evaluated on limited time series of SR data (starting 1998). (**a**) Environmentally-sensitive Beverton-Holt, (**b**) environmentally-sensitive Ricker, (**c**) classic Beverton-Holt, (**d**) classic Ricker, (**e**) hockey-stick function (the latter fitted on the limited time series). Increasingly darker colors in (a) and (b) correspond to predictions with the 5^th^, 50^th^ and 95^th^ percentile of historic North Sea SST

**
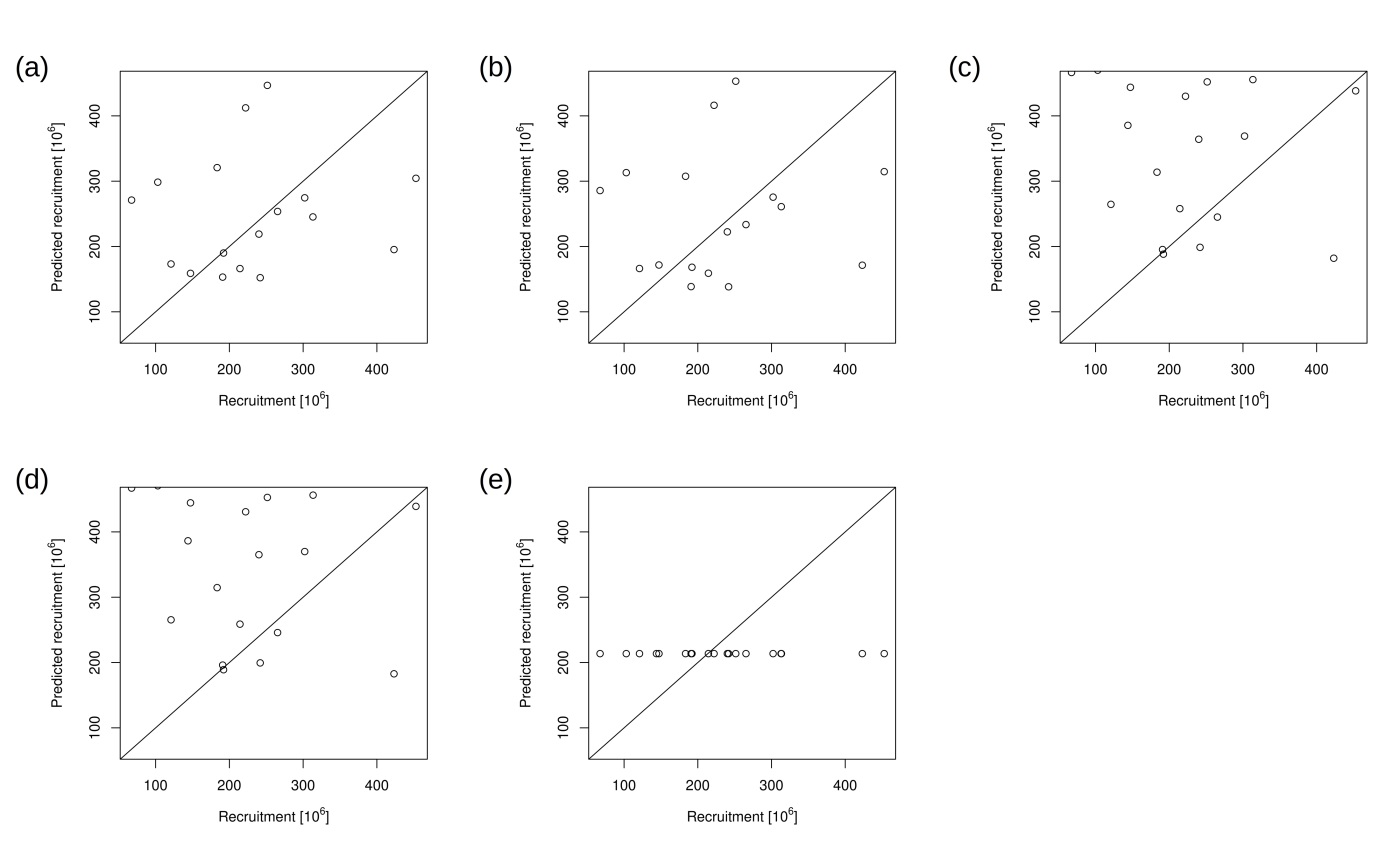
**

**Supplementary Figure M7.4.** Relationship between predicted recruitment and official recruitment estimates for environmentally-sensitive and –insensitive SR functions fitted on North Sea cod recruitment and SSB and North Sea SST, evaluated on limited time series of SR data (starting 1998). (**a**) Environmentally-sensitive Beverton-Holt, (**b**) environmentally-sensitive Ricker, (**c**) classic Beverton-Holt, (**d**) classic Ricker, (**e**) hockey-stick function (the latter fitted on the limited time series)

**Supplementary Results 3: Comparison of single projections conducted with different functional forms of the SR relationship**

Projections conducted with the two functional forms of the SR relationship under equal levels of catch and equal climate scenario revealed temporally reduced levels of recruitment for the Ricker SR function compared to the Beverton-Holt SR function, and a stronger pattern of regularly increasing and decreasing SSB (Suppl. Fig. R3.1). These observations are likely founded in the negative effect of relatively high levels of SSB on recruitment strength in the Ricker model, leading to a periodic increase and decrease of population size.


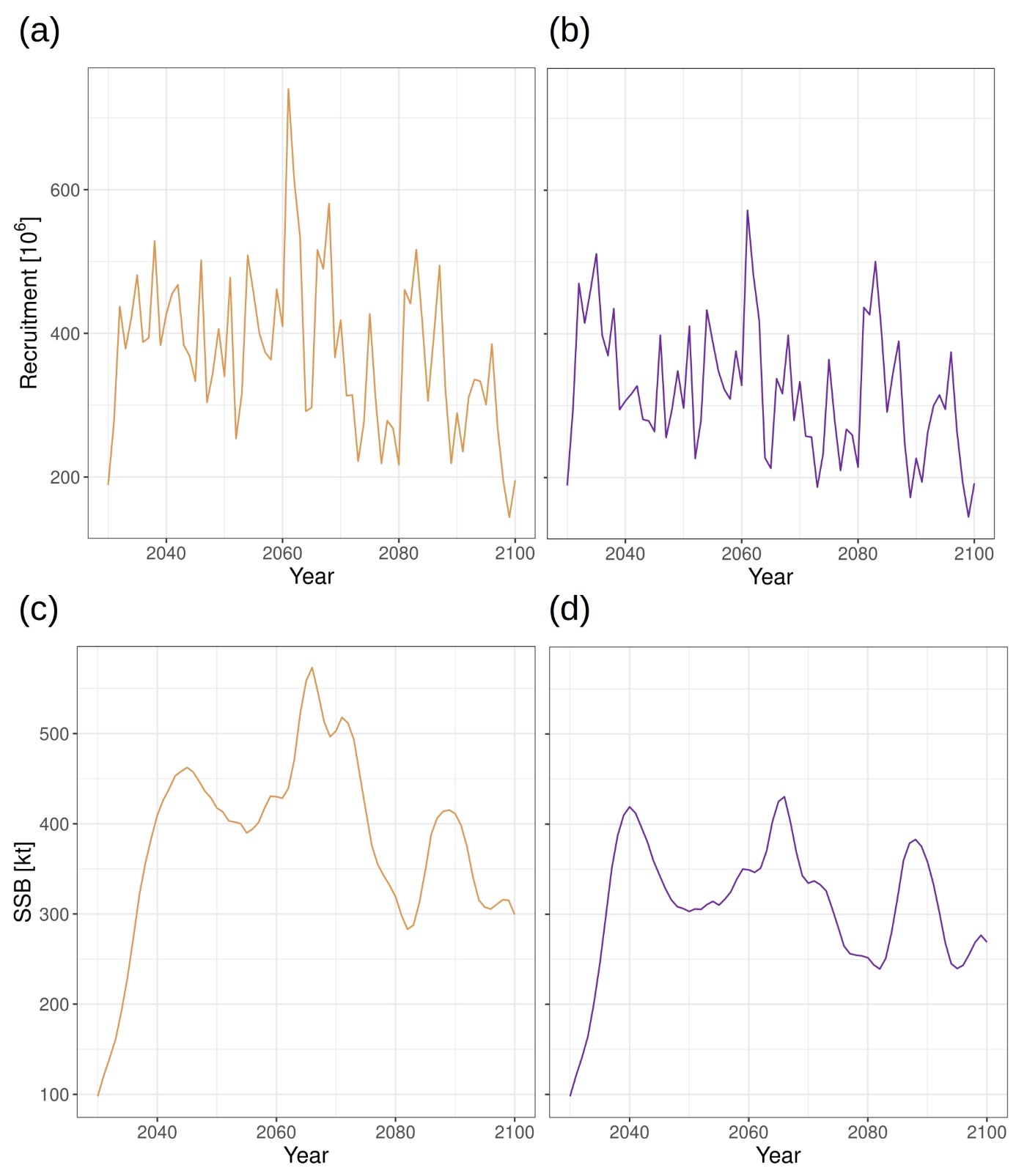


**Supplementary Figure R3.1.** Projections of recruitment (**a**, **b**) and SSB (**c**, **d**) conducted with a Beverton-Holt- (**a**, **c**) and a Ricker SR function (**b**, **d**). Projections were conducted with the mean parameter estimates for the SR functions (see Tab. 1 in the main text), under a catch level of 30 kt and under the RCP4.5 climate scenario

**Supplementary References**

[1] Allen, R. L. Models for fish populations: a review. *New Zeal. Oper. Res.*, **4,** 1-20 (1975)

[2] Baranov, F. I. On the question of the biological basis of fisheries. *Nauch. Issledov. Iktiol. Inst. Izv.*, **1,** 81-128 (1918)

[3] Lindegren, M., Checkley Jr., D. M., Rouyer, T., MacDall, A. D. and Stenseth, N. C. Climate, fishing, and fluctuations of sardine and anchovy in the California Current. *PNAS*, **110,** 13672-13677; 10.1073/pnas.1305733110 (2013)

[4] Pope, J. G. An investigation of the accuracy of virtual population analysis using cohort analysis. *ICNAF Res. Bull.*, **9,** 65-74 (1972)

[5] ICES. Cod (*Gadus morhua*) in Subarea 4, Division 7.d, and Subdivision 20 (North Sea, eastern English Channel, Skagerrak). ICES Working Group on the Assessments of Demersal Stocks in the North Sea and Skagerrak, **3 (66),** 79-162; 10.17895/ices.pub.8211 (2021)

[6] Mohn, R. The retrospective problem in sequential population analysis: An investigation using cod fishery and simulated data. *ICES J. Mar. Sci.*, **56,** 473-488; 10.1006/jmsc.1999.0481 (1999)

[7] Beverton, R. J. H. and Holt, S. J. *On the Dynamics of Exploited Fish Populations*. (Chapman & Hall, 1957); 10.1007/978-94-011-2106-4

[8] Hilborn, R., and Walters, C. J. *Quantitative Fisheries Stock Assessment. Choice, Dynamics and Uncertainty*. 570 pp. (Chapman and Hall, 1992); 10.1007/978-1-4615-3598-0

[9] Ricker, W. E. Stock and recruitment. *J. Fish. Res. Board Can.*, **11,** 559-623; 10.1139/f54-039 (1954)

[10] Ricker, W. E. Computation and interpretation of biological statistics of fish populations. *Bull. Fish. Res. Board Can.*, **191**; 10.2307/3800109 (1975)

[11] Elzhov, T. V., Mullen, K. M., Spiess, A.-N., and Bolker, B. 2016. minpack.lm: R interface to the Levenberg-Marquardt nonlinear least-squares algorithm found in MINPACK, plus support for bounds. URL: <https://CRAN.R-project.org/package=minpack.lm>. (2016). Last access on 15^th^ June, 2023

[12] Peck, M. A. *et al.* Climate change and European fisheries and aquaculture. CERES Project Synthesis Report. 110 pp (Universität Hamburg, 2020); 10.25592/uhhfdm.804

[13] Huang, B. *et al.* Extended Reconstructed Sea Surface Temperature, Version 5 (ERSSTv5): upgrades, validations, and intercomparisons. *J. Clim.*, **30,** 8179-8205; 10.1175/JCLI-D-16-0836.1 (2017)

[14] Maraun, D. Bias correcting climate change simulations – a critical review. *Curr. Clim. Change Rep.*, **2,** 211-220; 10.1007/s40641-016-0050-x (2016)

[15] Schenk, H., Zimmermann, F. and Quaas, M. The economics of reversing fisheries-induced evolution. *Nat. Sustain*., **6,** 706-711; 10.1038/s41893-023-01078-9 (2023)

[16] BLE. Monatsbericht 2020. Bericht über die Fischerei und die Marktsituation für Fischereierzeugnisse in der Bundesrepublik Deutschland. 49 pp. (German federal office for agriculture and food [BLE], 2020)

[17] ICES. Cod (27.47d20). Benchmark Workshop on North Sea Stocks (WKNSEA). *ICES Scientific Reports*, **3 (25),** 5-46; 10.17895/ices.pub.7922 (2021)
